# CXCL12^+^ fibroblastic reticular cells in lymph nodes facilitate immune tolerance by regulating T cell–mediated alloimmunity

**DOI:** 10.1172/JCI182709

**Published:** 2025-05-01

**Authors:** Yuta Yamamura, Gianmarco Sabiu, Jing Zhao, Sungwook Jung, Andy J. Seelam, Xiaofei Li, Yang Song, Marina W. Shirkey, Lushen Li, Wenji Piao, Long Wu, Tianshu Zhang, Soyeon Ahn, Pilhan Kim, Vivek Kasinath, Jamil R. Azzi, Jonathan S. Bromberg, Reza Abdi

**Affiliations:** 1 Transplantation Research Center and; 2 Renal Division, Brigham and Women’s Hospital, Harvard Medical School, Boston, Massachusetts, USA.; 3 Institute for Genome Sciences, University of Maryland School of Medicine, Baltimore, Maryland, USA.; 4 Department of Surgery and; 5 Center of Vascular and Inflammatory Diseases, University of Maryland School of Medicine, Baltimore, Maryland, USA.; 6 R&D Division, IVIM Technology, Seoul, South Korea.; 7 Graduate School of Medical Science and Engineering,; 8 Korea Advanced Institute of Science and Technology Institute for Health Science and Technology, and; 9 Graduate School of Nanoscience and Technology, Korea Advanced Institute of Science and Technology, Daejeon, South Korea.

**Keywords:** Immunology, Transplantation, Adaptive immunity, Tolerance

## Abstract

Fibroblastic reticular cells (FRCs) are the master regulators of the lymph node (LN) microenvironment. However, the role of specific FRC subsets in controlling alloimmune responses remains to be studied. Single-cell RNA sequencing (scRNA-Seq) of naive and draining LNs (DLNs) of heart-transplanted mice and human LNs revealed a specific subset of CXCL12^hi^ FRCs that expressed high levels of lymphotoxin-β receptor (LTβR) and are enriched in the expression of immunoregulatory genes. CXCL12^hi^ FRCs had high expression of CCL19, CCL21, indoleamine 2,3-dioxygenase (IDO), IL-10, and TGF-β1. Adoptive transfer of ex vivo–expanded FRCs resulted in their homing to LNs and induced immunosuppressive environments in DLNs to promote heart allograft acceptance. Genetic deletion of *LTβR* and *Cxcl12* in FRCs increased alloreactivity, abrogating the effect of costimulatory blockade in prolonging heart allograft survival. As compared with WT recipients, CXCL12^+^ FRC–deficient recipients exhibited increased differentiation of CD4^+^ T cells into Th1 cells. Nano delivery of CXCL12 to DLNs improved allograft survival in heart-transplanted mice. Our study highlights the importance of DLN CXCL12^hi^ FRCs in promoting transplant tolerance.

## Abstract 2

CXCL12+FRCs facilitated transplant tolerance. The delivery of CXCL12 by encapsulation into nanoparticles, leading to immune suppressive environments in lymph nodes and improved graft survival rates.

## Graphical abstract

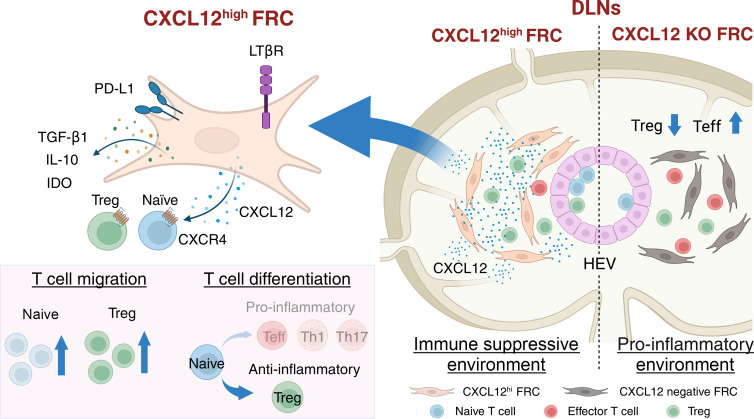

## Introduction

Lymph nodes (LNs) are the critical sites at which alloimmunity is activated. We have shown that the microenvironment within the LNs is a major determinant of the equilibrium between alloimmunity and suppression of the alloimmune response (1–4). Specific activities within the cellular and stromal compartments of the LNs determine the properties of the alloimmune response. Fibroblastic reticular cells (FRCs) constitute a major subset of LN stromal cells (LNSCs). Recent data from our research indicate that T cells enter LNs in distinct ways depending on whether tolerance or immunity is being established, and they localize to specific domains within LNs (2, 3, 5–9). FRCs are crucial for supporting the integrity of structures inside LNs, including high endothelial venules (HEVs) and the LN capsule, and thereby controlling the homing of immune cells into and within LNs (10, 11). Notably, FRCs play a pivotal role in producing chemokines such as CCL19, which promote the homing of T cells across HEVs (12). Once inside LNs, T cells migrate along extracellular matrix (ECM) fiber conduits constructed by FRCs to interact with DCs, localize to discrete domains, or egress from the LNs (13). These critical activities by FRCs are essential to the contact between T cells and antigen-presenting cells, an interplay central to allorecognition (12, 14–17). Our studies have shown that FRCs play an important role in tolerance induction, through recruitment of naive T cells to differentiate into inducible Tregs (iTregs) under the costimulatory blockade (3, 6, 18–20). FRCs play a crucial role in maintaining immune homeostasis by expressing various immunomodulatory molecules such as indoleamine 2,3-dioxygenase (IDO), arginase, and programmed death-ligand 1 (PD-L1) (12, 21). The immunosuppressive properties of FRCs can inhibit T cell responses and promote tolerance, contributing to the prevention of autoimmunity and excessive immune reactions (12). In contrast, the immunostimulatory properties of FRCs can support immune activation and effector functions, facilitating effective immune responses against pathogens or cancer cells (22, 23). These multifaceted functions of FRCs provide the capacity to control Th cell differentiation toward either pathogenic alloreactive or immunoregulatory cells. Understanding the complex determinants of these polarized FRCs is essential for developing targeted therapies to manipulate immune responses in different disease contexts (23, 24).

Single-cell genomics has revealed a multitude of LNSC subsets, each playing unique and critical roles in immune responses and tissue homeostasis (25–27). We have utilized single-cell RNA sequencing (scRNA-Seq)to study the heterogeneity within LNs; this has uncovered the potential role of LNSCs in modulating tolerance, especially the effect of costimulatory blockade on FRCs (3, 19, 20). However, a major gap in current understanding is the functional roles of distinct FRC subsets and their heterogeneity at the single-cell level within the draining LNs (DLNs) after transplantation. In addition, the role of these specific FRC subsets in controlling transplant tolerance within DLNs remains to be elucidated. By delineating the molecular and functional differences between immunosuppressive and immunostimulatory FRC subsets at a single-cell resolution, we can gain deeper insights into their specific roles in immune regulation.

Lymphotoxin-β receptor (LTβR) signaling in FRCs plays crucial roles in the development and maintenance of LNs (28, 29) The deletion of LTβR in FRCs causes a reduction in LN size with lower cellularity and an impaired conduit network (19, 28, 30). In addition, LTβR-dependent maturation of FRCs results in optimal activation of the T cell–mediated immune response in LNs during viral infection (28). These previous findings led us to speculate that LTβR^+^ FRCs are crucial for transplant tolerance induction, acting by regulating T cell–mediated immunity. Given the heterogeneity of FRC subsets and their distinct roles in immune regulation, delving deeper into the subsets of FRCs is crucial to a better understanding of the various immunoregulatory functions of those subsets that can substantially influence the outcome of transplant tolerance.

In this report, we identified a subset of immunoregulatory FRCs that coexpress LTβR and CXCL12. These LTβR^+^CXCL12^+^ FRCs facilitate T cell migration to DLNs and constrain T cell immunity by inducing T cell differentiation toward immunosuppressive phenotypes, thereby prolonging heart allograft survival. Furthermore, nanodelivery of CXCL12 to LNs improved graft survival after heart transplantation.

## Results

### Adoptive transfer of FRCs promotes immunosuppressive environment in DLNs.

**Figure 1:**
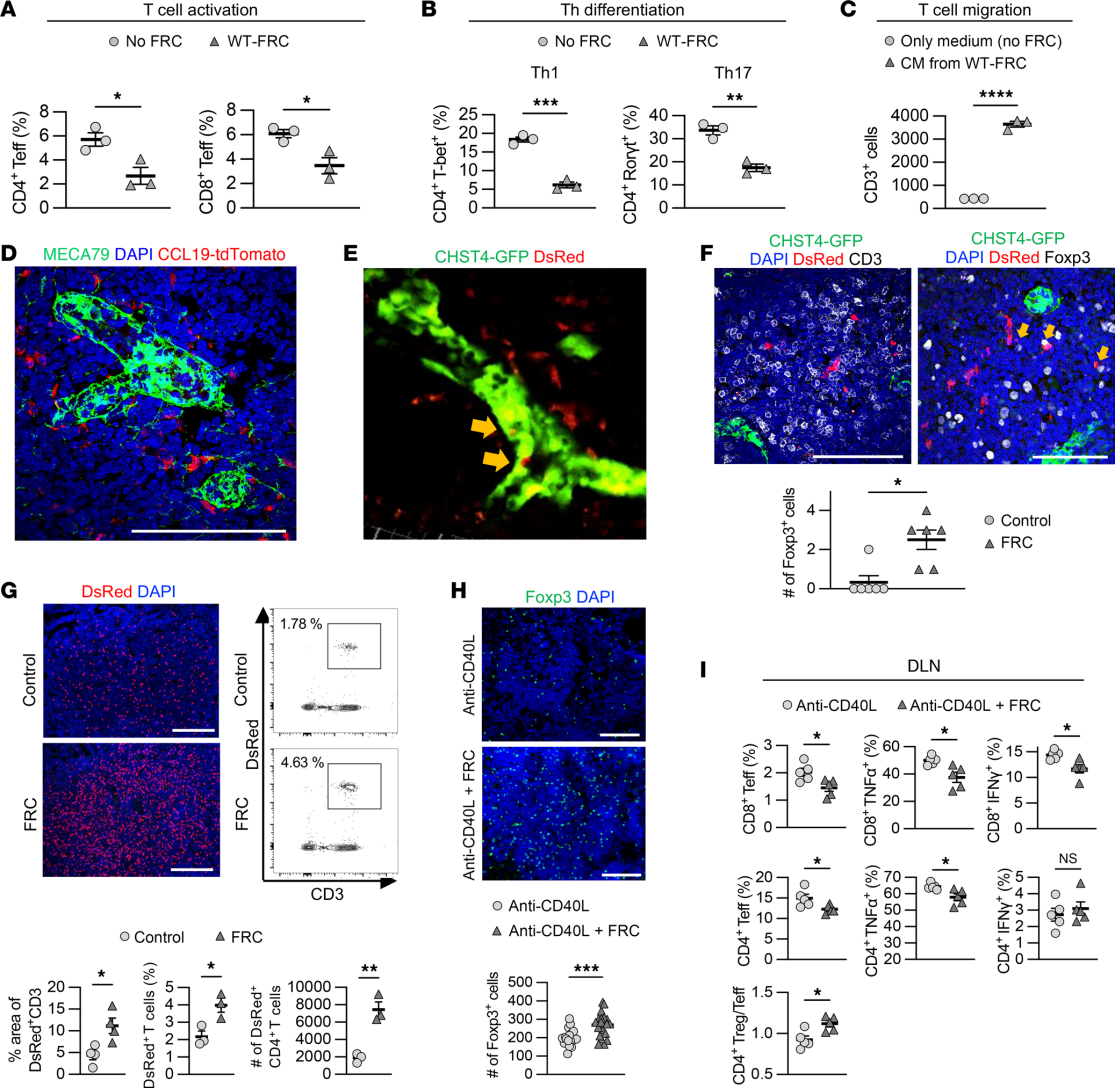
Adoptive transfer of FRCs promotes immune regulatory environment in DLNs. (**A**) T cell activation assay of CD3^+^ T cells cocultured with or without WT-FRCs. Percentage of effector T cells evaluated by flow cytometry. (**B**) Th differentiation assay of CD4^+^ T cells cocultured with or without WT-FRC administration. Percentage of CD4^+^T-bet^+^ cells, and CD4^+^Rorγt^+^ cells evaluated by flow cytometry. (**C**) Chemotaxis assay of T cells with CM from cultured WT-FRCs. (**D**) Representative images of LNs injected with FRCs from *Ccl19^Cre^*tdTomato mice. Scale bars: 100 μm. (**E**) Intravital imaging of LNs injected with DsRed^+^ FRCs into *Chst4*-GFP mice. Yellow arrows indicate injected FRCs inside HEVs. (**F**) Representative images of LNs injected with FRC from DsRed mice. Yellow arrows indicate the FRCs contacting Foxp3^+^ cells. Scale bars: 100 μm. (**G**) Representative images and comparison of injected DsRed^+^ T cell homing in LNs with or without FRCs (*n* = 3–4 mice/group). (**H**) Representative images and comparison of Foxp3-stained DLNs from WT C57BL/6J recipients with or without FRCs after heart transplantation (*n* = 5 mice/group). Scale bars: 100 μm. (**I**) Flow cytometry analysis of DLNs from WT C57BL/6J recipients with or without FRC administration after heart transplantation (*n* = 5 mice/group). Student’s *t* test for comparisons between 2 groups. Data are represented as means ± SEM. *P < 0.05; **P < 0.01; ***P < 0.001; ****P < 0.0001.

To investigate the effect of FRCs on the T cell alloimmune response, we isolated FRCs from WT C57BL/6J mice (WT-FRCs) and performed in vitro assays for T cell activation and Th differentiation, coculturing T cells with FRCs. Coculture of T cells with FRCs reduced CD4^+^ and CD8^+^ effector T cell, Th1, and Th17 differentiation in flow cytometry (Figure 1, A and B). Moreover, conditioned medium (CM) from cultured WT-FRCs promoted CD3^+^ T cell migration in a Transwell assay (Figure 1C).

Next, we sought to determine whether adoptively transferred FRC home to LNs and prolong heart allograft survival in WT C57BL/6J mice. We isolated primary FRCs from *Ccl19^Cre^*tdTomato mice and DsRed mice and administered them into naive WT C57BL/6J mice (2.0 × 10^5^ cells, i.v., 3 times, every 24 hours). We also utilized carbohydrate sulfotransferase 4-GFP (*Chst4*-GFP) mice to visualize the HEVs. Both *Ccl19^Cre^*tdTomato FRCs and DsRed FRCs were detected adjacent to HEVs in the LN parenchyma (Figure 1, D and E). Injected FRCs were also observed within HEVs of *Chst4*-GFP mice (Figure 1E) as well as within the vicinity of Foxp3^+^ Tregs (Figure 1F). To investigate the impact of exogenous FRCs on T cell homing to LNs, we adoptively transferred DsRed^+^ T cells into WT C57BL/6J mice with or without FRC treatment. Immunofluorescent (IF) staining and flow cytometry of LNs revealed increased trafficking of DsRed^+^ T cells to LNs of FRC-treated mice as compared with controls (Figure 1G). We also assessed the distribution of adoptively transferred FRCs in various organs outside the LNs. While DsRed^+^ FRCs were detected in the lung vasculature within 24 hours after injection, these cells had nearly disappeared by 48 hours after injection. Very few injected FRCs were detected in other organs, such as the liver and kidney (Supplemental Figure 1A; supplemental material available online with this article; https://doi.org/10.1172/JCI182709DS1). We also investigated the difference between the distribution of injected FRCs to the DLNs of heart-transplanted mice as compared with LNs of naive mice. DsRed^+^ FRCs were more numerous in the allograft DLNs than in the naive LNs (Supplemental Figure 1B). We also examined the surface expression of adhesion molecules. We found that FRCs express several adhesion molecules and chemokine receptors, including ICAM1, VCAM1, CD44, and CCR2 (Supplemental Figure 1C).

Heart allografts from BALB/c mice were then transplanted into WT C57BL/6J mice, and recipients were treated with anti-CD40L (125 μg, i.v., on day 0 after transplantation) (*n* = 4), FRCs alone (*n* = 4), or anti-CD40L plus FRCs (*n* = 9). FRC treatment (2.0 × 10^5^ cells, i.v., on days –1, 1, 3, and 5 after transplantation) synergized with anti-CD40L and significantly prolonged heart allograft survival compared with treatment with FRCs alone (*P* = 0.002, median survival time [MST] 25 days versus 8.5 days) or anti-CD40L alone (*P* = 0.02, MST, 25 days versus 11.5 days) (Supplemental Figure 1D). IF staining of heart allografts showed less interstitial fibrosis and fewer F4/80^+^ macrophages in recipients treated with anti-CD40L and FRCs compared with those treated with anti-CD40L alone (Supplemental Figure 1E). Collagen deposition was also decreased (Supplemental Figure 1E), and Foxp3 expression was elevated in the DLNs of recipients treated with anti-CD40L and FRCs compared with anti-CD40L alone (Figure 1H). Flow cytometry of DLNs showed that mice treated with anti-CD40L and FRCs exhibited fewer activated CD4^+^ and CD8^+^ T cells, including CD4^+^ effector T cells, CD8^+^ effector T cells, CD4^+^TNFα^+^ cells, CD8^+^TNFα^+^ cells, and CD8^+^IFNγ^+^ cells and a higher CD4^+^ Treg/T effector ratio, compared with anti-CD40L–treated mice (Figure 1I). These results suggested that WT-FRCs facilitate transplant tolerance by inducing an immunosuppressive environment with reduced fibrosis and inflammatory T cells and increased Foxp3^+^ cells in DLNs.

Lastly, we isolated and expanded FRCs from the LNs of MHC class II KO mice and investigated the effect of their transfer on heart graft survival. Similar to WT-FRCs, MHC class II–KO-FRCs also improved heart allograft survival following anti-CD40L treatment (*P* = 0.01, 22 days versus 11.5 days) (Supplemental Figure 1D).

### LTβR^+^ signaling in FRCs is required for transplant tolerance.

LTβR signaling in FRCs is critical to the development and maintenance of LNs (28, 31). Our recent report demonstrated that *Ccl19^Cre^Ltbr^fl/fl^* mice had collapsed fibroblastic networks and decreased conduits in LNs (19). To further explore the role of LTβR^+^ FRCs in LNs, we evaluated naive *Ccl19^Cre^Ltbr^fl/fl^* mice in which there was specific deletion of LTβR in FRCs (Supplemental Figure 2). In their naive state, *Ccl19^Cre^Ltbr^fl/fl^* mice had fewer LN CD4^+^, CD8^+^ T cells, and CD11c^+^ DCs compared with WT C57BL/6J mice. *Ccl19^Cre^Ltbr^fl/fl^* mice showed increased activation of T cells and a reduced LN CD4^+^ Treg/T effector (Teff) ratio (Supplemental Figure 3A). We also examined the homing of adoptively transferred T cells from DsRed mice and DCs from CD11c-GFP mice. *Ccl19^Cre^Ltbr^fl/fl^* mice exhibited reduced migration of both T cells and DCs to DLNs compared with WT C57BL/6J mice (Supplemental Figure 3, B and C). FRC administration enhanced DC homing to LNs in WT mice and partially rescued the impaired DC migration in *Ccl19^Cre^Ltbr^fl/fl^* mice (Supplemental Figure 3C). Analysis of DC subsets revealed no significant differences in the proportions of conventional type 1 DCs (cDC1s), conventional type 2 DCs (cDC2), and plasmacytoid DCs (pDCs) between WT and *Ccl19^Cre^Ltbr^fl/fl^* mice (Supplemental Figure 3D).

**Figure 2:**
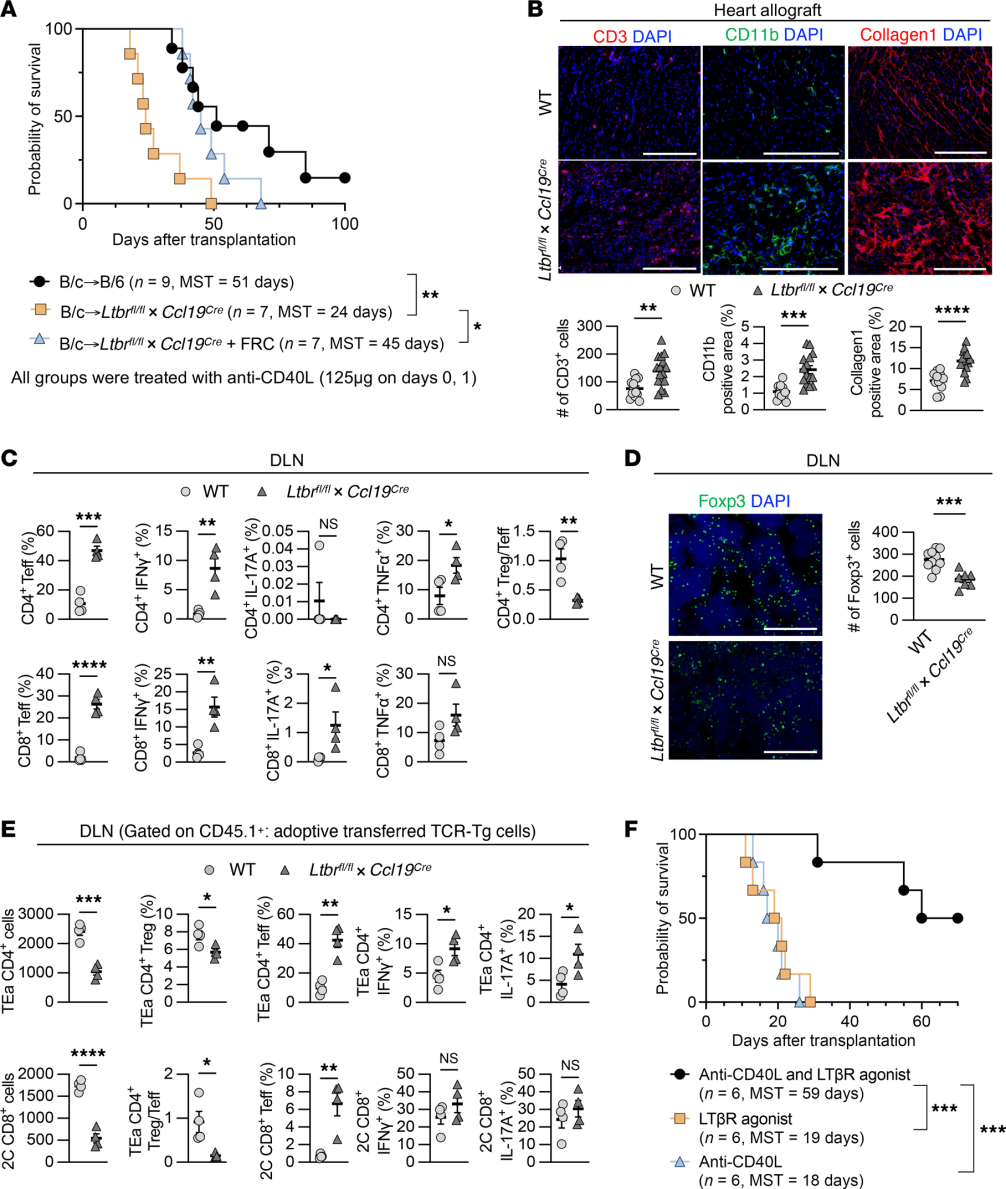
Deletion of LTβR^+^ signaling on FRCs abrogates transplant tolerance. (**A**) Comparison of heart allograft survival among WT C57BL/6J, *Ccl19^Cre^Ltbr^fl/fl^*, and *Ccl19^Cre^Ltbr^fl/fl^* recipients with FRC injections (*n* = 7–9 mice/group). Graft survival data were combined from 3 independent experiments. (**B**) Representative images and comparison of CD3-, CD11b-, and collagen 1–stained heart allografts from WT C57BL/6J and *Ccl19^Cre^Ltbr^fl/fl^* recipients (*n* = 4 mice/group). Scale bars: 200 μm. (**C**) Flow cytometry analysis of DLNs from WT C57BL/6J and *Ccl19^Cre^Ltbr^fl/fl^* recipients (*n* = 4 mice/group). (**D**) Representative images and comparison of Foxp3-stained DLNs (*n* = 4 mice/group). Scale bars: 200 μm. (**E**) Flow cytometry analysis of transferred TCR Tg cells in DLNs (*n* = 4 mice/group). (**F**) Comparison of heart allograft survival among WT C57BL/6J recipients treated with anti-LTβR agonist mAb alone (20 μg i.v. on day 0), anti-CD40L alone (40 μg i.v. on day 0), and both anti-LTβR agonist mAb and anti-CD40L of BALB/c hearts (*n* = 6 mice/group). Graft survival data were combined from 2 independent experiments. log-rank test for graft survival. Student’s *t* test for comparisons between 2 groups. Data are represented as means ± SEM. *P < 0.05; **P < 0.01; ***P < 0.001; ****P < 0.0001.

Next, we investigated the effect of LTβR signaling in FRCs on transplant tolerance. As compared with WT recipients, *Ccl19^Cre^Ltbr^fl/fl^* mice had impaired heart allograft survival (*P* = 0.002, 51 days versus 24 days, respectively) (Figure 2A). Heart allografts in *Ccl19^Cre^Ltbr^fl/fl^* mice contained denser immune cell infiltrates, including CD44^+^CD62L^–^ effector T cells, proinflammatory cytokine-producing T cells, mature DCs, and macrophages, more severe interstitial fibrosis, and more occluded vasculature as compared with those in WT C57BL/6J mice (Figure 2B, Supplemental Figure 3E, and Supplemental Figure 4A). Flow cytometry analysis of DLNs showed that *Ccl19^Cre^Ltbr^fl/fl^* recipients had more effector T cells with lower CD4^+^ Treg/Teff ratios (Figure 2C). IF staining showed fewer Foxp3^+^ cells and exhibited markedly increased formation of Ki67^+^ germinal centers (GCs) in the DLNs of the *Ccl19^Cre^LTtbr^fl/fl^* mice compared with WT mice (Figure 2D and Supplemental Figure 4B).

To evaluate alloantigen-specific T cell responses, we adoptively transferred I-E^d^–specific T cell receptor transgenic (TCR Tg) TEa CD4^+^ cells and H-2L^d^–specific TCR Tg 2C CD8^+^ cells, each recognizing BALB/c donor-specific alloantigens, into WT C57BL/6J and *Ccl19^Cre^Ltbr^fl/fl^* mice. Heart allografts from BALB/c donors were transplanted 24 hours after the cell transfer, and DLNs were collected and analyzed 4 days after the transplantation, gating on the transferred cells (CD45.1^+^) (Supplemental Figure 5). Flow cytometry revealed that a greater number of TEa CD4^+^ cells and 2C CD8^+^ cells homed to the DLNs of WT C57BL/6J mice compared with *Ccl19^Cre^Ltbr^fl/fl^* mice (Figure 2E). Examining the activation markers of T cells, we found that the DLNs of the *Ccl19^Cre^Ltbr^fl/fl^* mice contained a greater number of activated TCR Tg T cells with a shift toward fewer Tregs and a lower Treg/Teff ratio compared with those from WT C57BL/6J mice (Figure 2E). These results indicated that the absence of LTβR signaling in FRCs reduces antigen-specific T cell migration but promotes T cell activation, resulting in a proinflammatory microenvironment in DLNs after heart transplantation.

To determine whether enhancing LTβR signaling would improve graft survival, we investigated the effect of the anti-LTβR agonistic mAb on transplant tolerance after heart transplantation. Anti-LTβR mAb (20 μg, i.v., on day 0 after transplantation) improved heart allograft survival synergistically with very low dose anti-CD40L (40 μg, i.v., on day 0 after transplantation) in comparison with either anti-LTβR alone (*P* = 0.0005, MST, 59 days versus 18 days) or anti-CD40L alone (*P* = 0.0005, MST, 59 days versus 19 days) (Figure 2F).

As shown in Figure 1, H and I, adoptively transferred WT-FRCs improved the LN microenvironment after heart transplantation by reducing fibrosis and inflammatory T cells and increasing Foxp3^+^ Tregs, thereby inducing an immunosuppressive environment. To determine the effect of WT-FRC transfer on the LN structure of *Ccl19^Cre^Ltbr^fl/fl^* mice and its effect on transplant tolerance, we isolated WT-FRCs and injected them into naive *Ccl19^Cre^Ltbr^fl/fl^* mice (1.0 × 10^5^ cells, once a week for total 5 weeks). Our data indicated that FRC injections restored the LN structure toward normal and increased Foxp3^+^cells in the LNs of *Ccl19^Cre^Ltbr^fl/fl^* mice (Supplemental Figure 6, A and B). Furthermore, FRC injections into *Ccl19^Cre^Ltbr^fl/fl^* mice also restored transplant survival, which was abrogated in *Ccl19^Cre^Ltbr^fl/fl^* mice (*P* = 0.01, MST= 45 days versus 24 days) (Figure 2A).

### Coexpression and regulation of LTβR and CXCL12 in FRCs.

**Figure 3:**
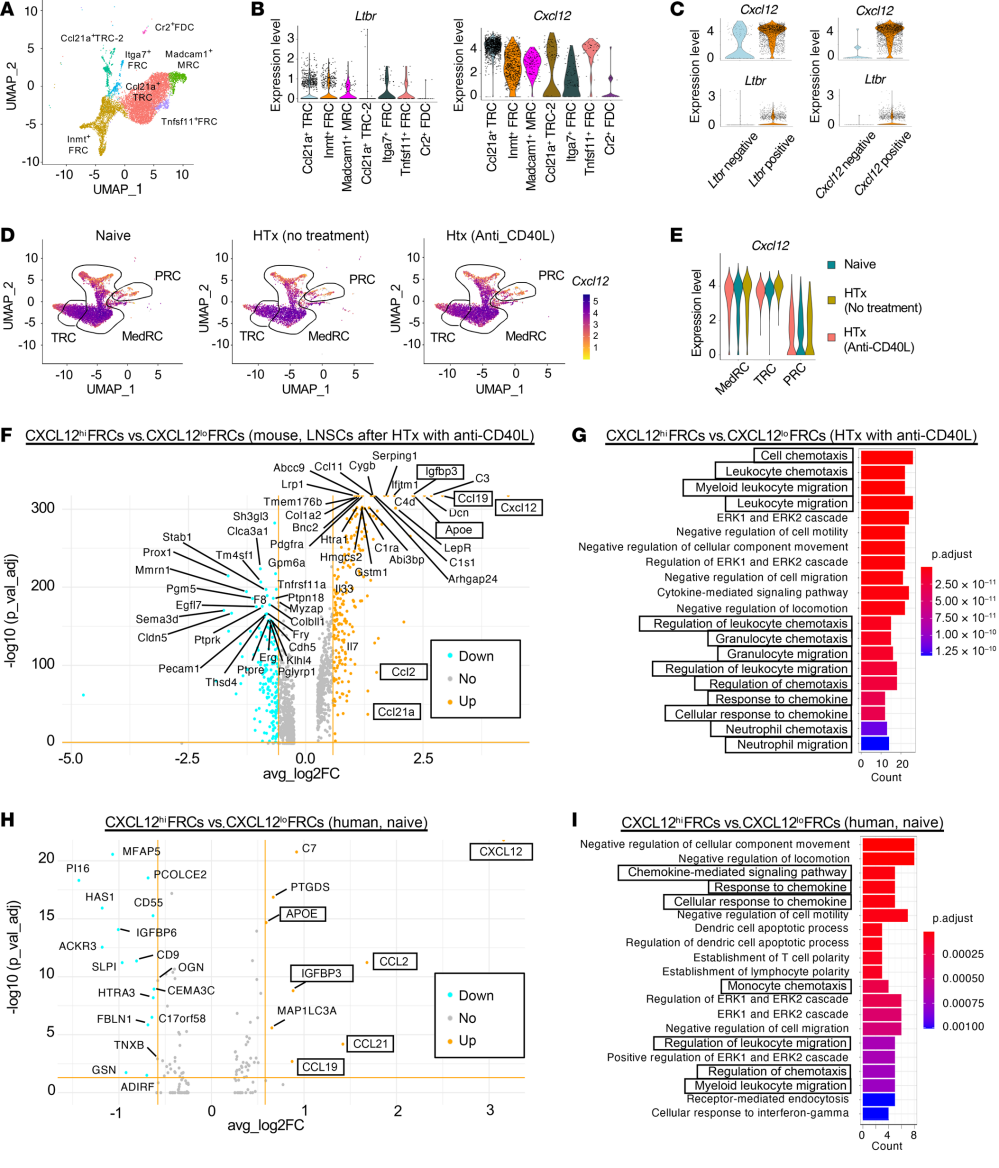
CXCL12^hi^ FRC subsets enriched for genes for T cell migration. (**A**) Unsupervised clustering of FRC subset clusters visualized with UMAP in LNSCs from naive LNs. (**B**) Violin plots of *Ltbr* and *Cxcl12* expression among FRC subset clusters in naive LNs. (**C**) Violin plots of *Ltbr* and *Cxcl12* expression in total FRCs from naive LNs. (**D**) UMAP of SC populations in naive LNs and DLNs, highlighting *Cxcl12* expression on MedRC, TRC, and PRC subsets. (**E**) Violin plots of *Cxcl12* expression in FRC subsets from naive LNs and DLNs. (**F**) Volcano plot comparing CXCL12^hi^ and CXCL12^lo^ FRCs in mouse LNSCs from heart transplanted recipients with anti-CD40L treatment. (**G**) Top 20 overrepresented ontology pathways based on DEGs in mouse LNSCs. (**H**) Volcano plot comparing CXCL12^hi^ and CXCL12^lo^ FRCs in human LNSCs. (**I**) Top 20 overrepresented ontology pathways in human LNSCs. Common genes shared between mouse and human are highlighted with bold letters surrounded by squares (**F** and **H**). Ontology pathways related to chemotaxis and migration are highlighted with bold letters surrounded by squares (**G** and **I**). HTx, heart transplantation.

Distinct subsets of FRCs perform various functions in regulating immune responses (12, 22–25, 32–34). LTβR activation has been shown to upregulate CXCL12 expression in blood endothelial cells (BECs) and lymphatic endothelial cells (LECs) (35, 36). To investigate the characteristics of LTβR^+^ and CXCL12^+^ FRCs, we analyzed scRNA-Seq data of LNSCs from naive LNs of WT C57BL/6J mice (19, 20). By unsupervised clustering, FRCs were divided into 7 subsets: Ccl21a^+^ T cell zone FRCs (TRCs), Inmt^+^ FRCs, Madcam1^+^ marginal reticular cells (MRCs), Ccl21a^+^ TRCs-2, Itga7^+^ FRCs, Tnfsf11^+^ FRCs, and Cr2^+^ follicular DCs (FDCs) (20, 25–27) (Figure 3A). Both *Ltbr* and *Cxcl12* were expressed in most subsets of FRCs, except for Cr2^+^ FDCs (Figure 3B). Cxcl12^+^ FRCs highly expressed *Ltbr*, and Ltbr^+^ FRCs had higher expression of *Cxcl12* compared with the negative populations (Figure 3C). *Ccl19^Cre^Ltbr^fl/fl^* mice showed decreased expression of CXCL12 in LNs (Supplemental Figure 7A). We also compared CXCL12 expression between LTβR^hi^ and LTβR^lo^ populations of FRCs by flow cytometry. The LTβR^hi^ population showed higher CXCL12 expression compared with the LTβR^lo^ population (Supplemental Figure 7B). Additionally, we evaluated the secretion of CXCL12 from isolated FRCs. The supernatant of FRC cultures from *Ccl19^Cre^Ltbr^fl/fl^* mice expressed less CXCL12 compared with that of isolated FRCs from WT C57BL/6J mice (Supplemental Figure 7C). LTβR agonist–treated WT-FRCs demonstrated a dose-dependent increase in CXCL12 secretion (Supplemental Figure 7D). These results indicated that LTβR signaling in FRCs regulates CXCL12 expression.

### CXCL12^+^ FRCs express genes that enrich T cell migration.

We first examined the distribution of CXCL12^hi^ FRCs in comparison with the other key FRC subsets in DLNs after transplantation, with or without anti-CD40L treatment. Our uniform manifold approximation and projection (UMAP) showed that TRCs, medullary FRCs (MedRCs), and perivascular reticular cells (PRCs) were the main cell populations encompassing high expression of *Cxcl12* transcripts (Figure 3D). The analysis of the proportion of each FRC subset from naive LNs and DLNs of transplant recipients untreated or treated with anti-CD40L showed that the percentage of the Ccl21a^+^ TRC population increased in anti-CD40L–treated heart transplant recipient mice as compared with other groups (Supplemental Figure 8A). Violin plots further showed that TRC and MedRC subsets had high mRNA expression of *Cxcl12* (Figure 3E and Supplemental Figure 8B). To characterize the microanatomical localization of CXCL12^hi^ FRCs after transplantation, we examined CXCL12 protein expression in DLNs from *Ccl19^Cre^*tdTomato mice by IF staining. In DLNs after transplantation, CXCL12 protein expression was upregulated mainly in FRC subsets of the T cell zone, around the HEV areas, and in the medullary area of the DLNs (Supplemental Figure 8C). However, analysis for differentially expressed genes (DEGs) showed that after anti-CD40L treatment, the downregulated genes in TRCs, MedRCs, and PRCs were primarily related to antigen presentation and T cell–mediated immunity (Supplemental Figure 8, D–F). These results suggested that the FRC subsets that upregulated CXCL12 after heart transplantation were reprogrammed by anti-CD40L for suppression of antigen presentation and T cell–mediated immunity.

Analysis of DEGs between CXCL12^hi^ and CXCL12^lo^ FRCs demonstrated higher expression of *Ccl19* and *Ccl21a* in CXCL12^hi^ FRCs (Figure 3F and Supplemental Figure 8G). CCL19 and CCL21a are crucial for the recruitment of naive T cells to LNs, which is a key step toward their differentiation to iTregs under costimulatory blockade (2, 3, 20). Ontology enrichGO analysis confirmed that CXCL12^hi^ FRC gene expression programs contribute to cell migration (Figure 3G). In addition, CXCL12^hi^ FRCs also showed higher expression of *Il-7* and *Il-33* (Figure 3F), which are related to Treg survival and stabilizing Treg molecular signatures, contributing to allograft tolerance (37, 38). To further confirm the characteristics of CXCL12^hi^ FRCs, we also performed a reanalysis of previously published human scRNA-Seq data of LNSCs (27). We divided the total FRC population, including CD266^+^ stromal cells (SCs), INMT^+^ SCs, CCL19^hi^ TRCs, CCL19^lo^ TRCs, and MRCs into 2 populations, CXCL12^hi^ and CXCL12^lo^, by defining the border at the lower quartile of *CXCL12* expression (Supplemental Figure 8G). Similar to the mouse scRNA-Seq data, CXCL12^hi^ FRCs in humans showed higher expression of *CCL19* and *CCL21* as well as enriched chemotaxis and migration-related genes (Figure 3, H and I).

### Ablation of CXCL12 in FRCs abrogates anti-CD40L–mediated transplant tolerance.

**Figure 4:**
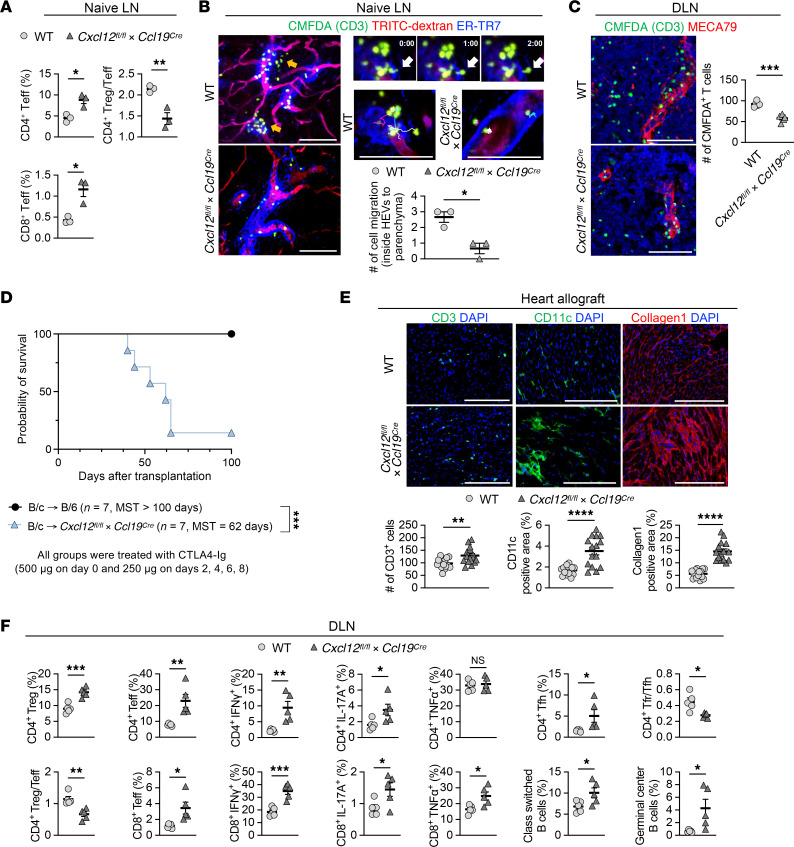
Ablation of CXCL12 of FRCs abrogates anti-CD40L mediated transplant tolerance. (**A**) Flow cytometry analysis of T cell compartments in naive LNs (*n* = 3 mice/group). (**B**) Representative intravital images of CMFDA-labeled CD3^+^ T cells injected into naive LNs. Yellow and white arrows indicate the migration of transferred CMFDA^+^ CD3^+^ T cells from inside HEVs into the parenchyma. Representative trajectories of transferred CD3^+^ T cells (gray, cyan, and magenta lines) and quantitative analysis of cell migration from inside HEVs toward the parenchyma for 20 minutes (*n* = 3). Scale bars: 100 μm. (**C**) In vivo T cell migration assay in a skin transplantation model. Representative images and quantitative analysis of CMFDA-labeled CD3^+^ T cells in DLNs (*n* = 5). Scale bars: 100 μm. (**D**) Comparison of heart allograft survival among the recipients under CTLA4 Ig treatment (*n* = 7 mice/group). Graft survival data were combined from 2 independent experiments. (**E**) Representative images and comparison of CD3-, CD11c-, and collagen 1–stained heart allografts (*n* = 5 mice/group). Scale bars: 200 μm. (**F**) Flow cytometry analysis of DLNs (*n* = 5 mice/group). log-rank test for graft survival. Student’s *t* test for comparisons between 2 groups. Data are represented as means ± SEM. *P < 0.05; **P < 0.01; ***P < 0.001; ****P < 0.0001.

To examine the functional role of CXCL12^+^ FRCs on transplant tolerance, we generated FRC-specific CXCL12-KO mice (*Ccl19^Cre^Cxcl12^fl/fl^* mice), in which there is specific deletion of CXCL12 in FRCs (Supplemental Figure 2). First, we compared naive WT C57BL/6J and *Ccl19^Cre^Cxcl12^fl/fl^* mice. *Ccl19^Cre^Cxcl12^fl/fl^* mice had higher levels of CD4^+^ and CD8^+^ effector T cells and lower Treg/Teff ratio in LNs (Figure 4A). Furthermore, the number of CD4^+^ and CD8^+^ T cells was reduced in naive LNs from *Ccl19^Cre^Cxcl12^fl/fl^* mice compared with those from WT mice (Supplemental Figure 9A). *Ccl19^Cre^Cxcl12^fl/fl^* mice also showed decreased migration of adoptively transferred CMFDA^+^CD3^+^ T cells to DLNs, compared with WT C57BL/6J mice both before and day 7 after skin allotransplantation (Figure 4, B and C). In addition, intravital imaging demonstrated that *Ccl19^Cre^Cxcl12^fl/fl^* mice had decreased migration from inside HEVs to parenchymal areas in naive LNs, compared with WT C57BL/6J mice (Figure 4B). These findings in *Ccl19^Cre^Cxcl12^fl/fl^* mice were similar to those in *Ccl19^Cre^Ltbr^fl/fl^* mice, suggesting the important relationship between LTβR and CXCL12 in FRCs (Supplemental Figure 3, A and B).

Next, to evaluate the effect of CXCL12 deletion in FRCs on transplant outcomes, we transplanted heart allografts from BALB/c donors to WT C57BL/6J or *Ccl19^Cre^Cxcl12^fl/fl^* recipients. All recipients were treated with CTLA4-Ig (500 μg i.p. on day 0 and 250 μg i.p. on days 2, 4, 6, and 8). WT C57BL/6J recipients showed long-term heart allograft survival as compared with *Ccl19^Cre^Cxcl12^fl/fl^* recipients (*P* = 0.0008, MST, 62 days compared with >100 days in WT C57BL/6J recipients) (Figure 4D). Immunohistochemistry of heart allografts showed more numerous CD3^+^ cells, CD11b^+^ cells, and CD11c^+^ cells in the hearts of *Ccl19^Cre^Cxcl12^fl/fl^* recipients. Interstitial fibrosis and C4d deposition were also higher in the heart allografts of *Ccl19^Cre^Cxcl12^fl/fl^* mice, as compared with WT C57BL/6J mice (Figure 4E and Supplemental Figure 9C). Flow cytometry of DLNs showed that *Ccl19^Cre^Cxcl12^fl/fl^* mice had more effector T cells as compared with WT C57BL/6J mice (Figure 4F). *Ccl19^Cre^Cxcl12^fl/fl^* mice also had a lower follicular regulatory T cell (Tfr)/follicular helper T cell (Tfh) ratio, more GC-like B cells, and more class-switched B cells in the DLNs (Figure 4F). We also investigated the infiltrating immune cells in heart allografts of anti-CD40L–treated *Ccl19^Cre^Cxcl12^fl/fl^* recipients as compared with anti-CD40L–treated WT recipients. Flow cytometry analysis of heart allograft infiltrates revealed that the number of proinflammatory cells was higher in *Ccl19^Cre^Cxcl12^fl/fl^* as compared with WT mice (Supplemental Figure 4). These results suggested that the lack of CXCL12 expressing FRCs in recipients increased alloreactivity, leading to worse graft survival.

We also investigated whether these CXCL12^+^ FRCs expanded after heart transplantation under anti-CD40L treatment. We found that CXCL12^+^ FRCs represented a higher proportion of the Ki67^+^ population in anti-CD40L–treated DLNs after heart transplantation, compared with nontreated DLNs after heart transplantation (Supplemental Figure 9D).

### CXCL12 increases CD4^+^ Treg migration.

**Figure 5:**
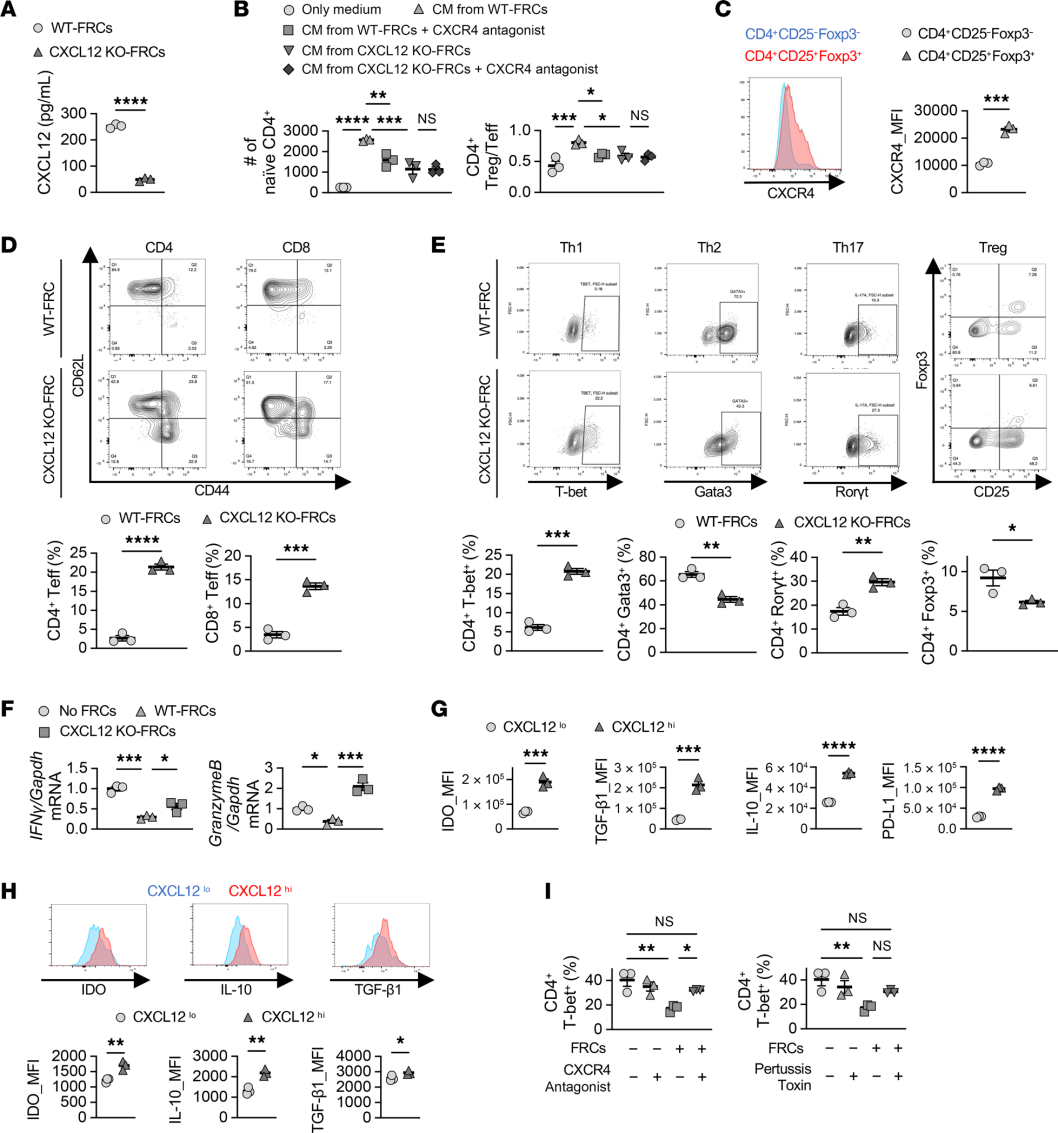
Deletion of CXCL12 in FRCs reduces Treg migration and immune suppressive effects for activated T cells. (**A**) Comparison of CXCL12 expression in supernatants of FRC culture 24 hours after incubation (*n* = 3/group). (**B**) Chemotaxis assay of CD4^+^ T cells toward CM from WT-FRCs or CXCL12-KO-FRCs (*n* = 3/group). (**C**) Comparison of CXCR4 expression between CD4^+^CD25^–^Foxp3^–^ and CD4^+^CD25^+^Foxp3^+^ T cells (*n* = 3/group). (**D**) T cell activation assay of CD3^+^ T cells cocultured with WT-FRCs or CXCL12-KO-FRCs (*n* = 3/group). (**E**) Differentiation of CD4^+^ T cells into Th1, Th2, Th17, and Treg subsets in the presence of WT-FRCs or CXCL12-KO-FRCs (*n* = 3/group). (**F**) mRNA expression of IFN-γ and granzyme b in CD3^+^ T cells cocultured with WT-FRCs or CXCL12-KO-FRCs (*n* = 3/group). (**G**) Comparison of IDO, TGF-β1, IL-10, and PD-L1 expression between CXCL12^lo^ and CXCL12^hi^ FRCs in mouse LNs (*n* = 3/group). (**H**) Comparison of IDO, IL-10, and TGF-β1 expression between CXCL12^lo^ and CXCL12^hi^ FRCs in human LNs (*n* = 3/group). (**I**) Flow cytometry analysis of CD4^+^ Th1 differentiation with or without FRCs. CD4^+^ T cells were pretreated with DMSO, CXCR4 antagonist, or pertussis toxin for 1 hour (*n* = 3/group). Student’s *t* test for comparisons between 2 groups. One-way ANOVA with Tukey’s multiple-comparisons test for multiple comparisons. Data are represented as means ± SEM. *P < 0.05; **P < 0.01; ***P < 0.001; ****P < 0.0001.

Our scRNA-Seq analysis suggested that CXCL12^hi^ FRCs facilitated chemotaxis. We therefore hypothesized that CXCL12 might contribute to the migration of naive T cells into LNs, promoting their conversion to Tregs under costimulatory blockade. We observed that CXCL12 secretion from CXCL12-KO-FRCs was significantly lower than that from WT-FRCs (Figure 5A). Next, we studied T cell migration using CM from cultured WT-FRCs and CXCL12-KO-FRCs. CM from WT-FRCs increased the migration of naive CD4^+^ T cells (CD4^+^CD44^-^CD62L^+^) compared with the CM from CXCL12-KO-FRCs. We also evaluated the balance of migrating cells between CD4^+^CD25^+^Foxp3^+^ Tregs and CD4^+^CD44^+^CD62L^–^ effector T cells. CM from WT-FRCs induced a higher Treg/Teff ratio of migrated cells, compared with CM from CXCL12-KO-FRCs (Figure 5B). CXCR4 is the key receptor for CXCL12. Treatment of T cells with a CXCR4 antagonist resulted in a significant decrease in migration to WT-FRC CM, compared with untreated T cells (Figure 5B). We also examined CXCR4 expression on CD4^+^CD25^+^Foxp3^+^ Tregs and non-Tregs (CD4^+^CD25^-^Foxp3^–^) in LNs. CXCR4 expression by Tregs was higher than non-Tregs in LNs (Figure 5C). These results suggested that FRCs contribute to the migration of T cells, especially Tregs, into LNs by secreting the chemokine CXCL12.

### CXCL12^hi^ FRCs possess a more immunosuppressive phenotype.

Next, we investigated the immunomodulatory function of CXCL12^+^ FRCs on T cell alloimmune responses by flow cytometry. Coculture of CD4^+^ and CD8^+^ T cells with CXCL12-KO-FRCs induced more effector T cells (CD44^+^CD62L^–^) compared with WT-FRCs (Figure 5D). Coculture of CD4^+^ T cells with CXCL12-KO-FRCs induced more differentiation to Th1 and Th17 cells and less differentiation to Th2 and Treg cells compared with WT-FRCs (Figure 5E). Coculture with WT-FRCs suppressed the mRNA expression of IFN-γ and granzyme b in activated CD3^+^ T cells. CXCL12 KO-FRCs had less of a suppressive effect on IFN-γ expression and even increased expression of granzyme b in T cells (Figure 5F).

Previous studies have shown that FRCs regulate T cell activation and differentiation through the expression of immunoregulatory cytokines, including IDO and TGF-β1 (15, 20, 39). We examined the expression of immunosuppressive molecules, including IDO, IL-10, TGF-β1, and PD-L1 by CXCL12^hi^ and CXCL12^lo^ populations in WT-FRCs. Flow cytometry analysis revealed that the CXCL12^hi^ population expressed higher levels of IDO, IL-10, TGF-β1, and PD-L1 compared with the CXCL12^lo^ population (Figure 5G).

We also evaluated CXCL12 expression in FRCs retrieved from human LNs and compared the expression of these immunosuppressive molecules between the CXCL12^hi^ and CXCL12^lo^ subsets. IF staining revealed CXCL12 expression in isolated FRCs from human LNs (Supplemental Figure 10A). Flow cytometry showed that approximately 80% of the FRC population expressed CXCL12 (Supplemental Figure 10B). Consistent with the mouse data, expression levels of IDO, IL-10, and TGF-β1 were higher in CXCL12^hi^ FRCs than CXCL12^lo^ FRCs (Figure 5H).

Next, we investigated the interaction between FRC-derived CXCL12 and its main receptor, CXCR4, on T cells (40). We pretreated CD4^+^ T cells with the CXCR4 antagonist AMD3100 or pertussis toxin, which inactivates diverse Gi/o G-protein–coupled receptors (41), and evaluated the suppressive effect of WT-FRCs on Th1 differentiation by flow cytometry. Both the CXCR4 antagonist and pertussis toxin reduced the suppressive effect of WT-FRCs, but they did not show any effect without FRCs (Figure 5I).

### Nanodelivery of CXCL12 to DLNs prolongs heart allograft survival.

**Figure 6:**
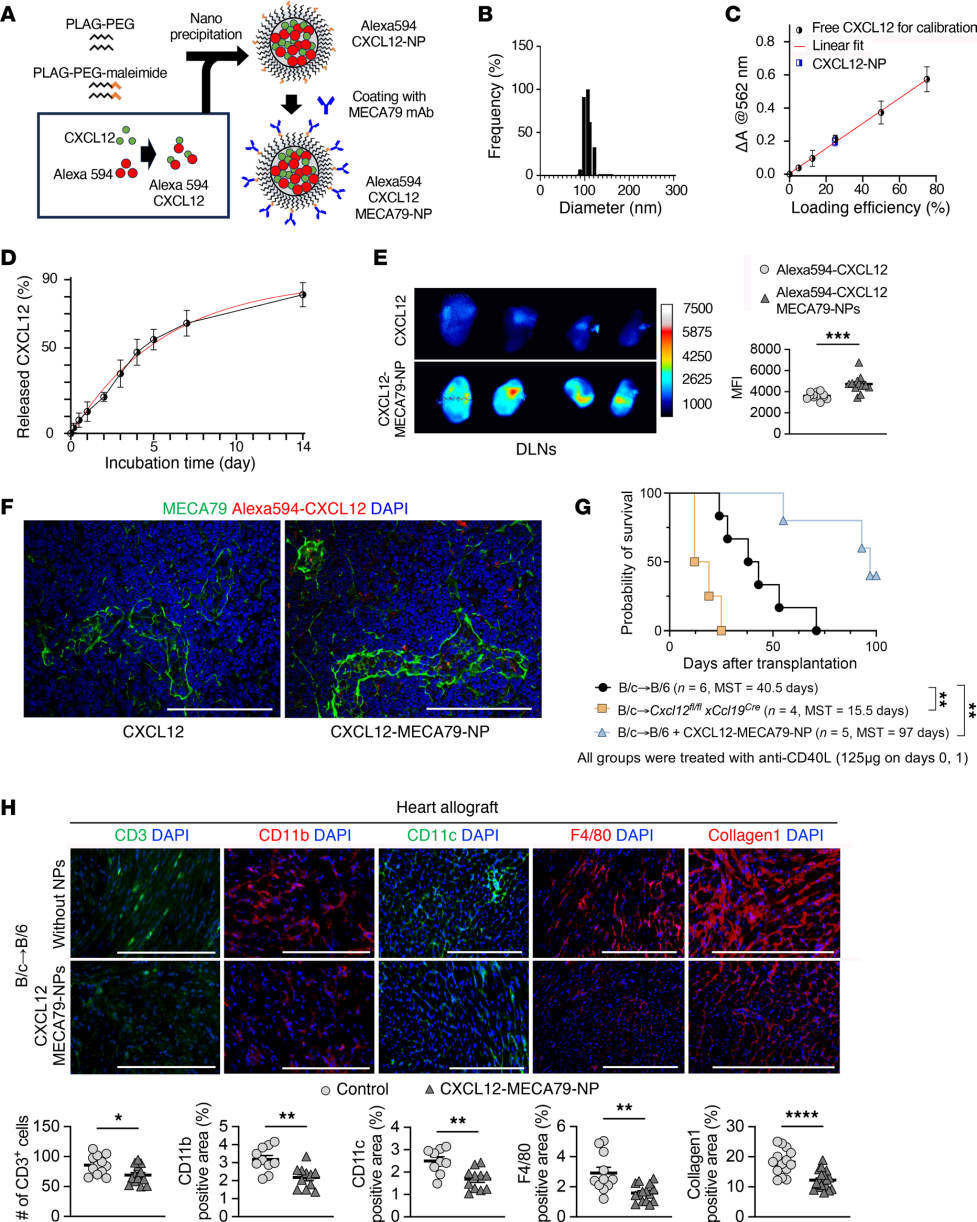
Nano delivery of CXCL12 to LNs prolongs heart allograft survival. (**A**) Schematic of Alexa Fluor 594–CXCL12–MECA79–NP synthesis. (**B**) Hydrodynamic size of MECA79-CXCL12-NPs in dynamic light-scattering analysis. (**C**) Loading efficiency of CXCL12 in NPs. (**D**) Release kinetics of CXCL12-NPs. (**E**) Ex vivo fluorescence imaging of DLNs from skin allograft recipients injected i.v. with free Alexa Fluor 594–CXCL12 or Alexa Fluor 594–CXCL12–MECA–79–NPs (*n* = 4 mice/group). (**F**) Representative images of DLNs 24 hours after i.v. injection of Alexa Fluor 594–CXCL12–MECA79–NPs. Scale bars: 100 μm (**G**) Comparison of heart allograft survival among recipient groups (*n* = 4–6 mice/group). (**H**) Representative images of heart allografts (*n* = 3 mice/group). Scale bars: 200 μm. log-rank test for graft survival. Student’s *t* test for comparisons between 2 groups. Data are represented as means ± SEM. *P < 0.05; **P < 0.01; ***P < 0.001.

Since CXCL12 appears to be integral for the suppressive effects of FRCs, we evaluated the effect of targeted nanodelivery of CXCL12 on allograft survival (Figure 6A). To deliver CXCL12 into DLNs efficiently, we encapsulated CXCL12 into poly (D, L-lactide-co-glycolide)-based (PLGA-based) nanoparticles (NPs), coated with MECA-79 antibody (CXCL12-MECA79-NPs) as previously reported (20, 42, 43). The hydrodynamic size of CXCL12-MECA79-NPs was around 90–120 nm, similar to empty NPs (Figure 6B) (20). The loading efficiency was approximately 25% (Figure 6C). Our in vitro kinetic assay showed that the release of CXCL12 from the NPs was sustained over 2 weeks (Figure 6D). We prepared Alexa Fluor 594–conjugated CXCL12 (Alexa Fluor 594–CXCL12) and Alexa Fluor 594–CXCL12 encapsulated into MECA-79-NPs (Alexa Fluor 594–CXCL12–MECA79–NPs). These were injected on day 7 after skin transplantation. At 24 hours after injection, ex vivo fluorescence imaging of DLNs showed that labeled CXCL12 more strongly accumulated in the DLNs from mice injected with Alexa Fluor 594–CXCL12–MECA79–NPs compared with those from mice injected with Alexa Fluor 594–CXCL12 without NP encapsulation (Figure 6E). IF staining also showed an accumulation of labeled CXCL12 in DLNs from Alexa Fluor 594–CXCL12–MECA79–NP–injected mice, mainly located inside HEVs and in the parenchyma around the HEVs (Figure 6F).

To evaluate the therapeutic effect, heart allografts from BALB/c donors were transplanted into WT C57BL/6J mice treated with anti-CD40L (125 μg i.v. on days 0 and 1) and with or without CXCL12-MECA79-NP treatment (5 μg i.v. on days –1, 0, 1, 2, and 3). Deletion of CXCL12 in FRCs shortened heart allograft survival as compared with WT recipients (*P* = 0.005, 15.5 days versus 40.5 days), while recipients treated with CXCL12-MECA79-NPs had prolonged heart allograft survival compared with those without CXCL12-MECA79-NP treatment (*P* = 0.007, 97 days vs 40.5 days) (Figure 6G). IF staining showed greater infiltration of inflammatory immune cells and markedly higher collagen 1 expression in heart allografts of the untreated control recipients than the CXCL12-MECA79-NP–treated recipients (Figure 6H). These results demonstrated that targeted delivery of CXCL12 to DLNs regulated the alloimmune response and prolonged allograft survival after heart transplantation.

## Discussion

We first investigated the homing of adoptively transferred FRCs into LNs. In *Chst4*-GFP mice, injected FRCs were observed within the HEV complex and accumulated in the surrounding HEV area, suggesting that FRCs might migrate into LNs via HEVs. Future real-time imaging of LNs following FRC injection could provide visualization of their trafficking and the steps FRCs undergo to transmigrate across HEVs. Previous studies demonstrated that CCR7-modified mesenchymal stem cells (MSCs) enhance the homing efficiency of transferred cells into LNs and attenuate the immune response during graft-versus-host disease (44). Our study revealed that FRCs express various adhesion molecules and chemokine receptors. Among those molecules, CD44 has been reported to promote the extravasation of activated T cells by binding to endothelial hyaluronan (45, 46). However, the precise roles of each adhesion molecule in the homing of FRCs to LNs remain to be fully elucidated.

A proinflammatory milieu within the DLNs drives LN remodeling (4, 12, 22, 47–50). Such structural and functional changes in LNs could interfere with DC and T cell interaction and the formation of iTregs under costimulatory blockade (12). Along with others, we have demonstrated that FRCs express a variety of immunoregulatory molecules, including IDO and PD-L1 (12, 39). Our research also shows that FRCs can modulate their microenvironment and regulate the composition of laminins, which may play a critical role in supporting Treg development and maintenance (2, 3, 19, 20). In this study, we demonstrated that the adoptive transfer of WT-FRCs following heart transplantation induces an immunosuppressive microenvironment, reduces fibrosis within DLNs, and promotes the homing of naive T cells into LNs. Taken together, these findings suggest that FRCs mediate immune tolerance through diverse direct and indirect mechanisms. FRCs are also known to express MHC class II, with its expression upregulated in response to inflammatory stimuli (51). However, we demonstrated that adoptive transfer of MHC class II KO-FRCs similarly improved heart allograft survival under anti-CD40L treatment. These results suggest that the immunoregulatory effects of FRCs are mediated through mechanisms independent of MHC class II.

In this study, we generated FRC-specific conditional KO mice using the constitutive *Ccl19-Cre* line, which is a well-established Cre model to study FRC biology (26, 28, 31, 52–55). Like many other conditional KO, the Cre-loxP system may potentially affect the CCL19^+^ population in other tissues; however, our findings suggest that the off-target deletion effects of LTβR and CXCL12 in other cell types, if any, are minimal.

LTβR signaling is key to the development and maturation of LNs (15, 22, 24, 28). In this study, we generated FRC-specific LTβR-KO mice, permitting a focus on LTβR expression selectively in FRCs. Using adoptively transferred allogeneic TCR-Tg T cells, we found that *Ccl19^Cre^Ltbr^fl/fl^* recipients had more activated alloreactive T cells for both MHC class I– and II–restricted antigens, including CD4^+^ effector T cells, CD8^+^ effector T cells, CD4^+^IFNγ^+^ T cells, and CD8^+^ IFNγ^+^ T cells compared with WT C57BL/6J recipients. These results revealed that lack of LTβR signaling in FRCs increased alloantigen-specific alloreactivity, abrogating the tolerogenic effects of costimulatory blockade. Furthermore, we demonstrated that enhancing LTβR signaling with LTβR agonism improved heart allograft survival, synergizing with the effects of costimulatory blockade. Considering the importance of LTβR signaling in FRCs on transplant tolerance, more efficient targeted delivery of LTβR agonists to FRCs in LNs might provide a therapeutic option for regulating immune responses in transplant recipients.

Previous studies have established the importance of LTβR signaling in FRCs for effective antiviral immune responses (28, 54). However, our findings demonstrate that *Ccl19^Cre^Ltbr^fl/fl^* mice had extensive alloreactive responses in a heart-transplantation model with anti-CD40L treatment. This discrepancy likely reflects fundamental differences between immune responses in infectious immunity and transplant alloimmunity. In our heart transplantation model treated with anti-CD40L, we observed that anti-CD40L therapy downregulated genes involved in antigen presentation and T cell–mediated immunity across different FRC subsets. However, the mechanisms of reprogramming by anti-CD40L treatment are complex and likely multifactorial. Generally, anti-CD40L targets CD40L-expressing activated T and B cells, thereby reducing their interaction with CD40^+^ antigen-presenting cells, such as DCs. This mechanism suppresses the infiltration of donor-reactive T cells, mitigates the humoral immune response, and promotes Treg induction (56–58). Considering the mechanism of FRC reprogramming by anti-CD40L, it is likely that FRCs are influenced not only by their interactions with CD40L^+^ cells but also by the state of CD40-expressing DCs, which are themselves affected by anti-CD40L–mediated inhibition. DCs are known to interact with FRCs and play a crucial role in regulating FRC biology via the CLEC2/PDPN axis (14, 16), further adding to the complexity of this mechanism.

Recent high-resolution analysis with confocal microscopy and scRNA-Seq revealed the topological and genetic heterogeneity of FRCs in LNs (12, 20, 22, 23, 25). Our scRNA-Seq of LNSCs showed coenrichment of *Ltbr* and *Cxcl12* expression in a specific subset of FRCs. LTβR signaling initiates both canonical and noncanonical NF-κB pathways, promoting gene transcription of cytokines, including *Ccl19* and *Ccl21*, and cell adhesion molecules (24, 59, 60). Previous studies have shown that LTβR stimulation upregulates CXCL12 expression via the noncanonical NF-κB pathway in BECs and LECs (35, 36, 61). Indeed, our in vitro study of FRCs also showed that LTβR signaling in FRCs contributes to the secretion of CXCL12. Building on this finding, we determined that deletion of either LTβR or CXCL12 in FRCs abrogated the effect of costimulatory blockade on allograft survival after heart transplantation, supporting the theory that LTβR signaling in FRCs improves allograft survival by inducing CXCL12.

CXCL12 is a homeostatic chemokine involved in the migration and proliferation of immune and hematopoietic cells (62–64). Our scRNA-Seq of LNSCs from anti-CD40L–treated DLNs after heart transplantation showed that CXCL12^hi^ FRCs expressed higher levels of *Ccl19* and *Ccl21* and exhibited enrichment in chemotaxis and cell migration–related genes compared with CXCL12^lo^ FRCs. In the T cell zone, CCL19 and CCL21 are key chemokines for CCR7^+^ naive T cell migration to LNs through HEVs, which is a key process for the formation of Tregs under anti-CD40L treatment after transplantation (12, 23, 65–68). Our intravital imaging and IF analysis of adoptively transferred T cells confirmed that genetic deletion of LTβR and CXCL12 in FRCs impaired T cell homing into LNs. Based on these results, we speculated that CXCL12^+^ FRCs facilitate transplant tolerance by promoting T cell trafficking to DLNs and suppressing T cell–mediated immunity after heart transplantation.

FRC-specific CXCL12-KO mice showed impaired T cell trafficking and abrogated the effect of costimulatory blockade on allograft survival after heart transplantation. Among FRC subsets, CXCL12 expression was upregulated mainly in TRCs, MedRCs, and PRCs after transplantation. TRCs are known to control cell interactions between T cells and DCs by guiding them and providing the appropriate stromal niche (69–73). FRCs in the T cell zone are reported to suppress T cell activation via IDO, adenosine 2A receptor, and TGFβR (39, 74). Indeed, both mouse and human CXCL12^hi^ FRCs expressed more immunosuppressive molecules, including IDO and IL-10, compared with the CXCL12^lo^ FRCs. Furthermore, CXCL12^hi^ FRCs also expressed higher levels of *Il-7* and *Il-33* compared with CXCL12^lo^ FRCs. TRCs and MRCs are known to express IL-7, which supports the survival and homeostasis of naive T cells (75, 76). IL-33 is primarily expressed by TRCs and MedRCs (77), and it helps to sustain Tregs (38, 78) as well as suppress proinflammatory Th1 cells (79). We found that CXCL12^+^ FRCs proliferated in DLNs after heart transplantation with anti-CD40L treatment, which may also contribute to the induction of tolerance by facilitating T cell trafficking and suppression of T cell immunity. These findings suggest that CXCL12^hi^ FRCs suppress T cell activation and induce transplant tolerance through a complex mechanism involving a variety of chemokines, cytokines, and immunosuppressive molecules.

Our study also demonstrated that the number of DCs in naive LNs was reduced in both *Ccl19^Cre^Ltbr^fl/fl^* and *Ccl19^Cre^Cxcl12^fl/fl^* mice compared with WT C57BL/6J mice. This finding was accompanied by impaired DC homing in *Ccl19^Cre^Ltbr^fl/fl^* *mice*, a phenomenon that was reversed by FRC administration. We postulate that FRC-mediated homing of DCs and naive T cells to LNs promotes the efficacy of anti-CD40L in converting naive T cells to iTregs. Tolerogenic DCs express a variety of regulatory molecules that can further potentiate Treg formation (80–82).

We demonstrated that *Ccl19^Cre^Cxcl12^fl/fl^* mice exhibited enhanced GC activity, as evidenced by increased frequencies of GL7^+^ GC B cells, IgD-negative class-switched B cells, and a lower Tfr/Tfh ratio. We postulate that Tfr cells might also be affected by the lack of CXCL12 expression in FRCs, potentially through the regulation of naive T cell homing or impaired immune cell regulation in LNs. Tfr cells play a pivotal role in modulating Tfh and B cell activation and suppressing GC responses (83–88). Future studies are warranted to examine the function of CXCL12^+^ FRCs in models of antibody-mediated rejection.

Transwell assays with CXCL12-KO-FRCs showed impaired T cell migration, especially of CD4^+^ Tregs, which have expressed higher levels of CXCR4 as compared with non-Tregs. In addition, treatment with CXCL12-MECA79-NP improved allograft survival, indicating the activity of CXCL12 in DLNs, especially in the vicinity of HEVs, on transplant tolerance. In a previous study, CXCL12 promoted the differentiation of Tregs and increased IL-10 expression in an experimental autoimmune encephalomyelitis model by TCR-stimulated human T cells (89–91). CXCL12 also attracted Tregs, facilitating the creation of an immunosuppressive microenvironment in a model of bone marrow transplantation (92, 93). The TCR associated with and transactivated CXCR4, promoting stabilization of cytokine mRNA transcripts, including *Il-2*, *Il-4*, and *Il-10*, in an in vitro study of human peripheral blood T cells stimulated through CD3 and CD28 (94). Both IL-4 and IL-10 have been shown to suppress cell-mediated immune responses (95, 96) and contribute to the differentiation of CD4^+^ T cells into Th2 cells (97–99). Taking these data together, the CXCL12-CXCR4 axis may contribute to FRC-mediated suppression of T cell–mediated immunity in DLNs and the protective effect of CXCL12^hi^ FRCs on allograft survival after heart transplantation through several regulatory mechanisms.

CXCL12 is an attractive therapeutic target for transplantation, as it has been used in other models (62, 100–102). CXCL12 is regulated by posttranslational modification, including proteolytic removal of NH2- or COOH-terminal amino acids. These modifications result in reduced or abrogated biological activity (62, 103). Therefore, an appropriate delivery system must be constructed to provide a sustained effect of CXCL12 treatment. Previous studies have demonstrated effective delivery of CXCL12 using NPs or scaffolds for bone repair by promoting the homing of adoptively transferred MSCs to the bone marrow (101, 102). Here, we demonstrated that a CXCL12-targeted delivery system using MECA79-NPs could localize efficiently to DLNs. This system can provide both sustained release of CXCL12 from the encapsulated NPs and escape from rapid degradation prior to its arrival at the DLNs, thereby enhancing the bioavailability of the chemokine. This modality of efficient CXCL12 delivery targeting DLNs might facilitate the recruitment of naive T cells into the DLNs and differentiation toward iTregs under costimulatory blockade, resulting in prolonged graft survival after heart transplantation.

We have established the critical role of CXCL12^hi^ FRCs in promoting transplant tolerance using a heterotopic heart-transplantation model. Applying similar strategies to other orthotopic transplantation models will be of significant interest.

In summary, we have demonstrated that the expression of both LTβR and CXCL12 by FRCs is important for promoting tolerance after heart transplantation. CXCL12^hi^ FRCs contribute to constraining T cell–mediated immunity and facilitating T cell differentiation toward immunosuppressive phenotypes. Therefore, targeted delivery of CXCL12 to LNs could support immune tolerance in transplantation.

## Methods

### Sex as a biological variable.

For studies involving humans and/or animal models, sex was not considered as a biological variable. Our study exclusively examined female mice to ensure data consistency and reduce variability among experimental groups. It is unknown whether the findings are relevant for male mice.

### Mice.

All experiments used weight-matched mice that were maintained in specific pathogen–free environments at 8–12 weeks of age.

Seven- to eight-week-old WT C57BL/6J (referred to as WT mice; stock 00064), BALB/cByJ (referred to as BALB/c mice; stock 001026), B6.Cg-Tg(CAG-DsRed*MST)1Nagy/J (referred to as DsRed mice; stock 006051), B6.Cg-Gt(ROSA)26Sortm14(CAG-tdTomato)Hze/J (referred to as RCL-tdT mice; stock 007914), B6.FVB-1700016L21RikTg (ItgaxDTR/EGFP)57Lan/J (CD11c-DTR/GFP, referred to as CD11c-GFP mice; stock 004509), B6.Cg-Tg(Chst4-EGFP)23Nrud/J (referred to as *Chst4*-GFP mice; stock 022787), and B6.129S2-H2^dlAb1-Ea^/J mice (referred to as MHC classIIKO mice; stock 003584) were purchased from The Jackson Laboratory. *Ccl19^Cre^* mice were a gift from Shannon Turley at Genentech (South San Francisco, California, USA). *Ccl19^Cre^* mice were crossed with the floxed allele of *Ltbr* or *Cxcl12* mice to generate *Ccl19^Cre^Ltbr^fl/fl^* mice or *Ccl19^Cre^Cxcl12^fl/fl^* mice. *Ccl19^Cre^* mice were crossed with RCL-tdT mice to generate *Ccl19^Cre^*tdTomato mice. Genotyping was performed by PCR, according to the protocol from The Jackson Laboratory. TCR Tg mice expressing the TEa TCR (recognizing I-Ed [Eα52-68] antigen in the context of I-Ab) (104) were provided by A.Y. Rudensky (Memorial Sloan Kettering Cancer Center, New York, USA). TCR Tg mice expressing the 2C TCR (recognizes the Ld class I MHC antigen SIYRYYGL peptide in the context of the H2b MHC class I molecule) (105) were provided by Thomas Gajewski (Ludwig Center for Cancer Research, University of Chicago, Chicago, Illinois, USA).

### Mouse heterotopic cardiac transplantation and skin transplantation.

Vascularized intraabdominal heterotopic heart transplantation was performed using microsurgical techniques, as previously described (20). WT C57BL/6J mice, *Ccl19^Cre^Ltbr^fl/fl^* mice, or *Ccl19^Cre^Cxcl12^fl/fl^* mice were transplanted with heterotopic heart allografts from BALB/c mice. As the standard protocol in this study, anti-CD40L mAb (MR1, 125 μg i.v. on days 0 and 1, BioXCell, BE0017-1) or CTLA4-Ig (Abatacept, 500 μg i.p. on day 0 and 250 μg i.p. on days 2, 4, 6, 8, Bristol-Myers Squibb) was administered to recipient mice. In the heart-transplantation model with FRC treatment, FRCs (2.0 × 10^5^ cells per injection, i.v. on days –1, 1, 3, and 5 after transplantation) and anti-CD40L (MR1, 125 μg i.v. on day 0) were administered. For the synergistic study of anti-LTβR agonist mAb with anti-CD40L mAb in the heart transplantation model, anti-LTβR agonist mAb (clone 3C8, 20 μg i.v. on day 0, Thermo Fisher Scientific, 16-5671-82) and anti-CD40L (MR1, 40 μg i.v. on day 0) were administered. The status of the heart allograft was monitored daily by abdominal palpation. Rejection was defined as the complete cessation of a palpable heartbeat, which was confirmed by direct visualization at laparotomy.

Mouse skin transplantation was performed, as previously described (4). Briefly, full-thickness skin was harvested from donor BALB/c mice. Skin allograft was cut into a 1 cm^2^ graft and sutured with 6-0 silk onto the upper back wounds of the recipient B6 mice. The skin grafts were protected using bandages until mice were sacrificed.

### Adoptive transfer of TCR-Tg T cells in heterotopic cardiac transplantation model.

TEa CD4^+^ T cells were isolated from CD45.1-TEa TCR-Tg mice using the CD4^+^ T Cell Isolation Kit, mouse (Miltenyi Biotec, 130-104-454). 2C CD8^+^ T cells were isolated from CD45.1-2C TCR-Tg mice using the CD8a (Ly-2) MicroBeads, mouse (Miltenyi Biotec, 130-117-044). TEa CD4^+^ and 2C CD8^+^ T cells (3.0 × 10^6^ cells each) were i.v. transferred into WT C57BL/6J mice or *Ccl19^Cre^Ltbr^fl/fl^* mice. Heterotopic heart allografts from BALB/c mice were transplanted 24 hours after transfer. At 96 hours after transplantation, DLNs were harvested, and the responses of transferred TCR-Tg T cells were analyzed by flow cytometry.

### FRC isolation from mouse LNs and human LNs.

Human iliac LNs were obtained from recipients of kidney transplants before receiving immunosuppression. FRCs from mouse and human LNs were isolated and purified as described previously (4). Details are available in Supplemental Methods.

### Evaluation of FRC trafficking in LNs and other organs.

The distribution of injected FRCs was evaluated by IF staining, intravital imaging, and flow cytometry. Details are available in Supplemental Methods.

### In vivo migration assay of DsRed^+^CD3^+^ T cells with WT C57BL/6J mice.

CD3^+^ T cells were isolated from the spleens of DsRed mice using the EasySep Mouse T Cell Isolation Kit (STEMCELL Technologics, 19851). For the FRC-treated groups, WT-FRCs (2.0 × 10^5^ cells per injection) were administrated i.v. 4 times, every other day. Twenty-four hours after the final FRC injection, 1.5 × 10^7^ DsRed^+^ T cells were injected i.v. At 12 hours after the injection of DsRed^+^ T cells, the distribution of injected DsRed^+^ T cells in the inguinal LNs, with and without FRC treatment, was evaluated by IF staining and flow cytometry.

### Immunohistochemistry and IF staining.

Immunohistochemistry and IF staining were performed with standard protocols. All images were captured using, ZEISS Axiolab5 (Carl Zeiss AG), the EVOS FL Auto 2 Imaging System (Thermo Fisher Scientific), or Athena Olympus FV3000 Confocal microscope (Olympus). For quantification, 3–5 random microscopic fields for each individual section were assessed. The percentages of positive area, the number of positive cells observed in low power fields, and the MFI were measured. Details are available in Supplemental Methods.

### Histological scoring.

Histological scoring of heart allografts was performed with a modified method from the International Society for Heart and Lung Transplantation (106, 107), as described previously (20, 42). Lymphocyte infiltration was graded from 0 to 4. The vascular score was evaluated by a combination of the vascular occlusion score and perivascular cellular infiltration. The vascular occlusion score was evaluated from grade 0 to 3 for every vessel. The perivascular cellular infiltration score was graded from 0 to 3.

### Flow cytometry.

Flow cytometry was performed with standard protocols. Details are available in Supplemental Methods.

### RNA analyses.

Total cellular RNA was isolated from primary cells using TRIzol Reagent (Thermo Fisher Scientific, 15596026) according to the manufacturer’s protocol. cDNA was synthesized using the iScript cDNA Synthesis Kit (Bio-Rad, 1708891). SYBR Green PCR Master Mix (Bio-Rad, 1725274) was used for quantitative PCR (qPCR). qPCR analysis was performed on the QuantStudio 3 Real-Time PCR System (Thermo Fisher Scientific). GAPDH was used as the internal controls. The ΔΔCT method was used to calculate relative gene expression of target genes with the internal controls. All primers used in this study are listed in Supplemental Table 1.

### scRNA-seq.

To assess the LNSCs at the transcriptional level after heart transplantation (day 8 after heart transplantation, anti-CD40L [250 μg, day 0]), we performed scRNA-Seq. Details are available in Supplemental Methods.

Seven FRC cell types from the previously published dataset (NCBI’s Gene Expression Omnibus database [GEO] GSE206837) were also used in this study (19). scRNA-Seq data for naive human LNSCs were also used from published data (E-MTAD-10206) (27). CXCL12 high/low subsets are defined based on the 1st quantile of expression of CXCL12. Genes with log_2_-fold change greater than 0.58 and FDR less than 0.05 are considered as significantly differential expressed. We used the R package “clusterProfiler” to perform GO overrepresentation analysis of DEGs to identify the enriched pathways between conditions. Enriched GO terms were identified with FDR of less than 0.05.

### In vivo migration assay with WT C57BL/6J mice and Ccl19^Cre^Cxcl12^fl/fl^ mice in skin transplantation model.

CD3^+^ T cells were isolated from the spleens of WT C57BL/6J mice using EasySep Mouse T Cell Isolation Kit and stained with CMFDA dye according to the manufacturer’s protocols (Thermo Fisher, C2925). To evaluate T cell trafficking into DLNs, 9.0 × 10^6^ CMFDA dye–stained CD3^+^ T cells were injected into WT C57BL/6J mice and *Ccl19^Cre^Cxcl12^fl/fl^* mice i.v. on day 8 after skin transplantation. At 2 hours after injections, DLNs were collected and evaluated by IF. The number of CMFDA^+^ T cells was counted in 1.6 mm^2^ of around HEVs area randomly.

### Intravital imaging of T cell homing into LNs from WT C57BL/6J mice and Ccl19^Cre^Cxcl12^fl/fl^ mice.

CMFDA dye–stained CD3^+^ T cells were prepared to evaluate T cell trafficking. To visualize HEVs in LNs, 40 μg Alexa Fluor 647–conjugated anti–ER-TR7 antibody (Santa Cruz Biotechnology, sc-73355 AF647) was injected intravenously into WT C57BL/6J mice and *Ccl19^Cre^Cxcl12^fl/fl^* mice 2 hours before imaging. Mice were anesthetized by i.p. injection of 100 mg/kg ketamine and 10 mg/kg xylazine. Then, 1.5 × 10^7^ CMFDA dye–stained CD3^+^ T cells and TRITC–Dextran (average mol wt 155,000 Da, 5 mg/mL, 100 μL; Sigma-Aldrich, T1287) dissolved in 1× PBS were injected 0.5 hours before imaging. The popliteal LNs were exposed surgically and confocal intravital imaging was performed (IVM-CMS3, IVIM Technology). Twenty-five *Z*-stack images were obtained with a 3 μm axial interval. Time-lapse images were obtained at a 1-minute time interval for 20 minutes with 5 sequential *Z*-stack images of 3 μm axial interval.

### Measurements of secreted CXCL12 from FRC.

Details are available in Supplemental Methods.

### Chemotaxis assay with conditional medium from cultured FRCs.

Details are available in Supplemental Methods.

### Evaluation of effects of FRCs on T cell activation and differentiation from naïve T cells.

Details are available in Supplemental Methods.

### Evaluation of the inhibition of CXCR4 and G protein–coupled receptor on Th1 differentiation.

Details are available in Supplemental Methods.

### Synthesis and characterization of NPs.

Details are available in Supplemental Methods.

### Evaluation of CXCL12-MECA79-NP distribution.

Details are available in Supplemental Methods.

### Statistics.

Data are expressed as means ± SEM. Unpaired 2-tailed *t* tests were used to assess the significance of comparisons between 2 groups, and 1-way ANOVA with post hoc Tukey’s multiple-comparisons test was used for comparison among more than 2 groups. A log-rank test was used for graft survival. *P* values of less than 0.05 were considered statistically significant for all comparisons.

### Study approval.

All animal experiments were approved by the IACUC of Brigham and Women’s Hospital (protocol 0167). The study protocol was reviewed and approved by the Institutional Review Board at the Brigham and Women’s Hospital and was conducted in full compliance with the principles of the Declaration of Helsinki. For the analysis of FRCs from human LNs, all patients provided written, informed consent. The study was approved by the Ethics Committees of the University of Maryland School of Medicine (IRB # HM-HP-00092098-6).

### Data availability.

The raw data of scRNA-Seq for LNSCs after heart transplantation have been deposited in the NCBI’s Gen Expression Omnibus database (GEO GSE262918). Values for all data points in graphs are reported in the Supporting Data Values file.

## Author contributions

YY designed and performed experiments, analyzed and interpreted data, and drafted the manuscript. GS performed experiments and interpreted data. JZ designed and performed experiments. SJ prepared NPs, performed experiments, and analyzed data. AJS, XL, MWS, LL, WP, LW, and TZ performed experiments. YS analyzed scRNA-Seq data. SA and PK performed intravital imaging and interpreted data. VK and JRA critically revised the manuscript. RA and JSB designed the study, interpreted the data, and critically revised and finalized the manuscript. The order of the co–first authors was determined by the time spent and their scientific contributions to the paper.

## Supplementary Material

Supplemental data

Supporting data values

## Version 1 05/01/2025

Electronic publication

## References

[1] Ochando JC (2005). Lymph node occupancy is required for the peripheral development of alloantigen-specific Foxp3+ regulatory T cells. J Immunol.

[2] Li L (2020). The lymph node stromal laminin α5 shapes alloimmunity. J Clin Invest.

[3] Li L (2022). Lymph node fibroblastic reticular cells preserve a tolerogenic niche in allograft transplantation through laminin α4. J Clin Invest.

[4] Li X (2020). Lymph node fibroblastic reticular cells deposit fibrosis-associated collagen following organ transplantation. J Clin Invest.

[5] Ochando JC (2006). Direct versus indirect allorecognition: Visualization of dendritic cell distribution and interactions during rejection and tolerization. Am J Transplant.

[6] Burrell BE (2012). Fates of CD4+ T cells in a tolerant environment depend on timing and place of antigen exposure. Am J Transplant.

[7] Burrell BE (2015). Lymph node stromal fiber ER-TR7 modulates CD4+ T cell lymph node trafficking and transplant tolerance. Transplantation.

[8] Simon T (2019). Differential regulation of T-cell immunity and tolerance by stromal laminin expressed in the lymph node. Transplantation.

[9] Brinkman CC (2016). Treg engage lymphotoxin beta receptor for afferent lymphatic transendothelial migration. Nat Commun.

[10] Novkovic M (2020). Topological structure and robustness of the lymph node conduit system. Cell Rep.

[11] Martinez VG (2019). Fibroblastic reticular cells control conduit matrix deposition during lymph node expansion. Cell Rep.

[12] Li L (2021). Lymph node fibroblastic reticular cells steer immune responses. Trends Immunol.

[13] Brown FD (2015). Fibroblastic reticular cells: organization and regulation of the T lymphocyte life cycle. J Immunol.

[14] Acton SE (2014). Dendritic cells control fibroblastic reticular network tension and lymph node expansion. Nature.

[15] Fletcher AL (2015). Lymph node fibroblastic reticular cells in health and disease. Nat Rev Immunol.

[16] Astarita JL (2015). The CLEC-2-podoplanin axis controls the contractility of fibroblastic reticular cells and lymph node microarchitecture. Nat Immunol.

[17] Perez-Shibayama C (2019). Fibroblastic reticular cells at the nexus of innate and adaptive immune responses. Immunol Rev.

[18] Warren KJ (2014). Laminins affect T cell trafficking and allograft fate. J Clin Invest.

[19] Li L (2023). FRC transplantation restores lymph node conduit defects in laminin α4-deficient mice. JCI Insight.

[20] Zhao J (2022). Delivery of costimulatory blockade to lymph nodes promotes transplant acceptance in mice. J Clin Invest.

[21] Keir ME (2008). PD-1 and its ligands in tolerance and immunity. Annu Rev Immunol.

[22] Lütge M (2021). Differentiation and activation of fibroblastic reticular cells. Immunol Rev.

[23] De Martin A (2024). Protective fibroblastic niches in secondary lymphoid organs. J Exp Med.

[24] Krishnamurty AT (2020). Lymph node stromal cells: cartographers of the immune system. Nat Immunol.

[25] Rodda LB (2018). Single-cell RNA sequencing of lymph node stromal cells reveals niche-associated heterogeneity. Immunity.

[26] Perez-Shibayama C (2020). Type I interferon signaling in fibroblastic reticular cells prevents exhaustive activation of antiviral CD8^+^ T cells. Sci Immunol.

[27] Kapoor VN (2021). Gremlin 1^+^ fibroblastic niche maintains dendritic cell homeostasis in lymphoid tissues. Nat Immunol.

[28] Chai Q (2013). Maturation of lymph node fibroblastic reticular cells from myofibroblastic precursors is critical for antiviral immunity. Immunity.

[29] Fütterer A (1998). The lymphotoxin beta receptor controls organogenesis and affinity maturation in peripheral lymphoid tissues. Immunity.

[30] Acton SE (2021). Communication, construction, and fluid control: lymphoid organ fibroblastic reticular cell and conduit networks. Trends Immunol.

[31] Cheng HW (2019). Origin and differentiation trajectories of fibroblastic reticular cells in the splenic white pulp. Nat Commun.

[32] Pikor NB (2021). Development and immunological function of lymph node stromal cells. J Immunol.

[33] Onder L (2022). Visualization and functional characterization of lymphoid organ fibroblasts. Immunol Rev.

[34] Takeuchi A (2018). A distinct subset of fibroblastic stromal cells constitutes the cortex-medulla boundary subcompartment of the lymph node. Front Immunol.

[35] Madge LA (2008). Lymphotoxin-alpha 1 beta 2 and LIGHT induce classical and noncanonical NF-kappa B-dependent proinflammatory gene expression in vascular endothelial cells. J Immunol.

[36] Piao W (2018). Regulation of T cell afferent lymphatic migration by targeting LTβR-mediated non-classical NFκB signaling. Nat Commun.

[37] Schmaler M (2015). IL-7R signaling in regulatory T cells maintains peripheral and allograft tolerance in mice. Proc Natl Acad Sci U S A.

[38] Kawai K (2021). IL-33 drives the production of mouse regulatory T cells with enhanced in vivo suppressive activity in skin transplantation. Am J Transplant.

[39] Knoblich K (2018). The human lymph node microenvironment unilaterally regulates T-cell activation and differentiation. PLoS Biol.

[40] Bianchi ME (2020). The chemokine receptor CXCR4 in cell proliferation and tissue regeneration. Front Immunol.

[41] Schneider OD (2009). Pertussis toxin signals through the TCR to initiate cross-desensitization of the chemokine receptor CXCR4. J Immunol.

[42] Bahmani B (2018). Targeted delivery of immune therapeutics to lymph nodes prolongs cardiac allograft survival. J Clin Invest.

[43] Jung S (2023). Nanotargeted delivery of immune therapeutics in type 1 diabetes. Adv Mater.

[44] Li H (2014). CCR7 guides migration of mesenchymal stem cell to secondary lymphoid organs: a novel approach to separate GvHD from GvL effect. Stem Cells.

[45] DeGrendele HC (1996). CD44 and its ligand hyaluronate mediate rolling under physiologic flow: a novel lymphocyte-endothelial cell primary adhesion pathway. J Exp Med.

[46] Siegelman MH (2000). The CD44-initiated pathway of T-cell extravasation uses VLA-4 but not LFA-1 for firm adhesion. J Clin Invest.

[47] Kasinath V (2019). Activation of fibroblastic reticular cells in kidney lymph node during crescentic glomerulonephritis. Kidney Int.

[48] Li X (2021). Kidney-draining lymph node fibrosis following unilateral ureteral obstruction. Front Immunol.

[49] Maarouf OH (2018). Repetitive ischemic injuries to the kidneys result in lymph node fibrosis and impaired healing. JCI Insight.

[50] Estes JD (2013). Pathobiology of HIV/SIV-associated changes in secondary lymphoid tissues. Immunol Rev.

[51] Malhotra D (2012). Transcriptional profiling of stroma from inflamed and resting lymph nodes defines immunological hallmarks. Nat Immunol.

[52] Gil-Cruz C (2016). Fibroblastic reticular cells regulate intestinal inflammation via IL-15-mediated control of group 1 ILCs. Nat Immunol.

[53] Perez-Shibayama C (2018). Fibroblastic reticular cells initiate immune responses in visceral adipose tissues and secure peritoneal immunity. Sci Immunol.

[54] Prados A (2021). Fibroblastic reticular cell lineage convergence in Peyer’s patches governs intestinal immunity. Nat Immunol.

[55] Cheng HW (2022). Intestinal fibroblastic reticular cell niches control innate lymphoid cell homeostasis and function. Nat Commun.

[56] Singh AK (2023). CD40-CD40L blockade: update on novel investigational therapeutics for transplantation. Transplantation.

[57] Kwun J (2024). The emerging era of organ transplantation and anti-CD154mAb. Am J Transplant.

[58] Elgueta R (2009). Molecular mechanism and function of CD40/CD40L engagement in the immune system. Immunol Rev.

[59] Dejardin E (2002). The lymphotoxin-beta receptor induces different patterns of gene expression via two NF-kappaB pathways. Immunity.

[60] Piao W (2021). LTβR signaling controls lymphatic migration of immune cells. Cells.

[61] Madge LA (2010). Classical NF-kappaB activation negatively regulates noncanonical NF-kappaB-dependent CXCL12 expression. J Biol Chem.

[62] Cambier S (2023). The chemokines CXCL8 and CXCL12: molecular and functional properties, role in disease and efforts towards pharmacological intervention. Cell Mol Immunol.

[63] Janssens R (2018). The unique structural and functional features of CXCL12. Cell Mol Immunol.

[64] Pawig L (2015). Diversity and inter-connections in the CXCR4 chemokine receptor/ligand family: molecular perspectives. Front Immunol.

[65] Hauser MA (2016). Common and biased signaling pathways of the chemokine receptor CCR7 elicited by its ligands CCL19 and CCL21 in leukocytes. J Leukoc Biol.

[66] Lo JC (2003). Differential regulation of CCL21 in lymphoid/nonlymphoid tissues for effectively attracting T cells to peripheral tissues. J Clin Invest.

[67] Marsland BJ (2005). CCL19 and CCL21 induce a potent proinflammatory differentiation program in licensed dendritic cells. Immunity.

[68] Mori S (2001). Mice lacking expression of the chemokines CCL21-ser and CCL19 (plt mice) demonstrate delayed but enhanced T cell immune responses. J Exp Med.

[69] Nakayama Y (2015). Murine fibroblastic reticular cells from lymph node interact with CD4+ T cells through CD40-CD40L. Transplantation.

[70] Bajénoff M (2006). Stromal cell networks regulate lymphocyte entry, migration, and territoriality in lymph nodes. Immunity.

[71] Schumann K (2010). Immobilized chemokine fields and soluble chemokine gradients cooperatively shape migration patterns of dendritic cells. Immunity.

[72] Mempel TR (2004). T-cell priming by dendritic cells in lymph nodes occurs in three distinct phases. Nature.

[73] Gasteiger G (2016). Lymph node - an organ for T-cell activation and pathogen defense. Immunol Rev.

[74] Lukacs-Kornek V (2011). Regulated release of nitric oxide by nonhematopoietic stroma controls expansion of the activated T cell pool in lymph nodes. Nat Immunol.

[75] Tan JT (2001). IL-7 is critical for homeostatic proliferation and survival of naive T cells. Proc Natl Acad Sci U S A.

[76] Onder L (2012). IL-7-producing stromal cells are critical for lymph node remodeling. Blood.

[77] Aparicio-Domingo P (2021). Fibroblast-derived IL-33 is dispensable for lymph node homeostasis but critical for CD8 T-cell responses to acute and chronic viral infection. Eur J Immunol.

[78] Faustino LD (2020). Interleukin-33 activates regulatory T cells to suppress innate γδ T cell responses in the lung. Nat Immunol.

[79] Tan Z (2021). IL-33/ST2 signaling in liver transplantation. Cell Mol Immunol.

[80] Li H (2015). Tolerogenic dendritic cells and their applications in transplantation. Cell Mol Immunol.

[81] Kenison JE (2024). Therapeutic induction of antigen-specific immune tolerance. Nat Rev Immunol.

[82] Bosteels V (2024). Striking a balance: new perspectives on homeostatic dendritic cell maturation. Nat Rev Immunol.

[83] Sage PT (2014). The coinhibitory receptor CTLA-4 controls B cell responses by modulating T follicular helper, T follicular regulatory, and T regulatory cells. Immunity.

[84] Clement RL (2019). Follicular regulatory T cells control humoral and allergic immunity by restraining early B cell responses. Nat Immunol.

[85] Mohammed MT (2021). Follicular T cells mediate donor-specific antibody and rejection after solid organ transplantation. Am J Transplant.

[86] Gassen RB (2022). T cell depletion increases humoral response by favoring T follicular helper cells expansion. Am J Transplant.

[87] Zhang H (2023). Transcriptionally distinct B cells infiltrate allografts after kidney transplantation. Transplantation.

[88] Wing JB (2014). Regulatory T cells control antigen-specific expansion of Tfh cell number and humoral immune responses via the coreceptor CTLA-4. Immunity.

[89] Meiron M (2008). CXCL12 (SDF-1alpha) suppresses ongoing experimental autoimmune encephalomyelitis by selecting antigen-specific regulatory T cells. J Exp Med.

[90] Karin N (2010). The multiple faces of CXCL12 (SDF-1alpha) in the regulation of immunity during health and disease. J Leukoc Biol.

[91] Kremer KN (2007). Haplotype-independent costimulation of IL-10 secretion by SDF-1/CXCL12 proceeds via AP-1 binding to the human IL-10 promoter. J Immunol.

[92] Dürr C (2010). CXCL12 mediates immunosuppression in the lymphoma microenvironment after allogeneic transplantation of hematopoietic cells. Cancer Res.

[93] Zou L (2004). Bone marrow is a reservoir for CD4+CD25+ regulatory T cells that traffic through CXCL12/CXCR4 signals. Cancer Res.

[94] Kremer KN (2017). TCR-CXCR4 signaling stabilizes cytokine mRNA transcripts via a PREX1-Rac1 pathway: implications for CTCL. Blood.

[95] Sieling PA (1993). Immunosuppressive roles for IL-10 and IL-4 in human infection. In vitro modulation of T cell responses in leprosy. J Immunol.

[96] Bromberg JS (1995). IL-10 immunosuppression in transplantation. Curr Opin Immunol.

[97] Kaplan MH (1996). Stat6 is required for mediating responses to IL-4 and for development of Th2 cells. Immunity.

[98] Takeda K (1996). Essential role of Stat6 in IL-4 signalling. Nature.

[99] Coomes SM (2017). CD4^+^ Th2 cells are directly regulated by IL-10 during allergic airway inflammation. Mucosal Immunol.

[100] Yu JR (2020). A liposome/gelatin methacrylate nanocomposite hydrogel system for delivery of stromal cell-derived factor-1α and stimulation of cell migration. Acta Biomater.

[101] Wang B (2018). Nanoparticle-modified chitosan-agarose-gelatin scaffold for sustained release of SDF-1 and BMP-2. Int J Nanomedicine.

[102] Chen Q (2018). Bone targeted delivery of SDF-1 via alendronate functionalized nanoparticles in guiding stem cell migration. ACS Appl Mater Interfaces.

[103] Janssens R (2018). Pathological roles of the homeostatic chemokine CXCL12. Cytokine Growth Factor Rev.

[104] Grubin CE (1997). Deficient positive selection of CD4 T cells in mice displaying altered repertoires of MHC class II-bound self-peptides. Immunity.

[105] Sha WC (1988). Selective expression of an antigen receptor on CD8-bearing T lymphocytes in transgenic mice. Nature.

[106] Billingham ME (1990). A working formulation for the standardization of nomenclature in the diagnosis of heart and lung rejection: Heart Rejection Study Group. The International Society for Heart Transplantation. J Heart Transplant.

[107] Stewart S (2005). Revision of the 1990 working formulation for the standardization of nomenclature in the diagnosis of heart rejection. J Heart Lung Transplant.

