# Supplementary material for: CXCL12+ fibroblastic reticular cells in lymph nodes facilitate immune tolerance by regulating T cell–mediated alloimmunity
Source: J Clin Invest. 2025 May 1;135(9):e182709. doi: 10.1172/JCI182709 (PMC12043101; doi:10.1172/JCI182709)
Supplement: Supplemental data [file jci-135-182709-s137.pdf]

Supplemental Methods

FRC isolation from mouse LNs and human LNs

Human iliac LNs were obtained from recipients of kidney transplants before receiving immunosuppression. FRCs from mouse and human LNs were isolated and purified as described previously (1). Collected mouse LNs or finely cut human LNs were digested in DMEM (Corning, Corning, NY, USA) containing 1.0 mg/mL collagenase D (Roche, Basel, Switzerland, 11088858001), 0.8mg/mL dispase II (Sigma-Aldrich, St.Louis, MO, USA, D4693-1G), and 0.1mg/mL DNase I (Roche, 10104159001) for 30 minutes at 37°C. This step was repeated one more time. All digested cells were collected. For flow cytometry, isolated FRCs were stained with the usual protocol and analyzed, focusing on the $CD45^{-}PDPN^{+}CD31^{-}$ population. For cell culture, isolated FRCs were cultured in DMEM containing 10% FBS (Lonza, Basel, Switzerland) and 1% penicillin/streptomycin (Cytiva, Marlborough, MA, USA). Only cells from passages 3-5 were used for experiments.

Evaluation of FRC trafficking in LNs and other organs

FRCs were isolated from DsRed mice and *Ccl19*^{Cre}tdTomato mice as described before. For the FRC trafficking assay to LNs and other organs by IF staining, FRCs from *Ccl19*^{Cre}tdTomato or DsRed mice were injected i.v. into WT C57BL/6J mice every day (3 times, 2.0 x 10^5 cells/each). The distribution of injected $DsRed^{+}$ FRCs in LN, lung, spleen, liver, kidney, heart, brain, and pancreas, was evaluated 24 hours and 48 hours after the last injection by IF staining. To evaluate the proximity of FRCs to Tregs, the number of $Foxp3^{+}$ cells within 50 µm of transferred FRCs or control points was quantified. Control points were randomly selected from areas away at least 100 µm from transferred FRCs. For intravital imaging, $DsRed^{+}$ FRCs were injected i.v. into WT C57BL/6J mice every day (3 times, 2.0 x 10^5 cells/each), and FRC trafficking in LNs was evaluated at 2 hours after the last injection.

To investigate the preferential distribution of ex vivo expanded FRCs into the DLNs of heart allograft, $DsRed^{+}$ FRCs were injected i.v. (2.0 x 10^5 cells/injections i.v. on days -1, 1, 3, and 5) and the number of $DsRed^{+}$ FRCs in the mediastinal LNs (naïve LNs and DLNs of heart allografts) was evaluated 24 hours after the last injection by flow cytometry.

Immunohistochemistry, Immunofluorescent staining

For immunohistochemistry, a part of heart allografts from each mouse was fixed in 10% buffered formalin (pH 7.2), embedded in paraffin, and sectioned at 5 μm. Hematoxylin and eosin (H&E) staining was performed with standard protocol. For IF staining, heart allografts and LNs were preserved in optimal cutting temperature (OCT) compound (Sakura Finetek, Torrance, CA, USA) and stored at –80°C. Tissues were cut into 8 μm sections, fixed in 4% PFA (Satna Cruz biotechnology, Dallas, TX, USA) for 5min at room temperature. Then, sections were blocked with 3% BSA (Sigma-Aldrich) in PBS (Corning) for 30 min at room temperature. Primary antibodies were diluted in blocking solution and incubated overnight at 4 ^{o}C or for 1 hour at room temperature. The secondary antibodies, conjugated to Alexa 488, 594 or 647 were incubated for 30 min at room temperature. DAPI (Thermo Fisher Scientific, Waltham, MA, USA) was used to stain the nuclei. For staining of *Ccl19*^{Cre}tdTomato, DsRed and *Chst4*-GFP, harvested tissues were fixed with 4% PFA at room temperature for 3 hours. Then, fixed tissues were treated with 30% sucrose buffer and preserved in OCT compound. The primary antibodies included rabbit anti-collagen1 polyclonal antibody (Bio-Rad, Hercules, CA, USA, 2150-1410), rat anti-CD11b monoclonal antibody (BioLegend, San Diego, CA, USA, 101201), rat anti-MECA79 monoclonal antibody (Santa Cruz Biotechnology, Dallas, TX, USA, sc-19602), rat anti-C4d monoclonal antibody (NOVUS Biologicals, Littleton, CO, USA, NB200-541), rat anti-Foxp3 monoclonal antibody (Thermo Fisher Scientific, 14-5773-82), rat anti-CD3 monoclonal antibody (Thermo Fisher Scientific, 14-0032-82), rat anti-F4/80 monoclonal antibody (Thermo Fisher Scientific, 14-4801-82), rat anti- ER-TR7 monoclonal antibody (Santa Cruz Biotechnology, sc73355), goat anti-Podoplanin polyclonal antibody (R&D Systems, Minneapolis, MN, AF3244), rat anti-Podoplanin monoclonal antibody (Thermo Fisher Scientific, 14-9381-82), goat anti-tdTomato polyclonal antibody (Biorbyt, Cambridge, UK, orb182397), mouse anti-CXCL12 monoclonal antibody (R&D Systems, MAB350), hamster anti-CD11c monoclonal antibody (Thermo Fisher Scientific, 16-0114-85), chicken anti-GFP polyclonal antibody (Thermo Fisher Scientific, A10262), rat anti-IgD monoclonal antibody (Thermo Fisher Scientific, 13-5993-82), rabbit anti-Ki67 monoclonal antibody

(NOVUS Biologicals, NB600-1252), rabbit anti-CCL19 polyclonal antibody (abcam, Cambridge, UK, ab221704).

Flow cytometry

DLNs were collected for flow cytometry. Single-cell suspensions were prepared by filtering the DLNs through a 70μm cell strainer. After the preparation of single-cell suspensions, cells were stained according to standard protocols. For intracellular cytokine staining, cells were first incubated with phorbol 12-myristate 13-acetase (PMA, 50 ng/mL) (MilliporeSigma, Burlington, MA, USA, 524400) and ionomycin (500 ng/mL) (MilliporeSigma, 407951) in combination with GolgiStop (BD Biosciences, Franklin Lakes, NJ, USA, 554724) at 37 °C for 4 hours. Then, these cells were stained with standard protocols. Flow cytometry was performed by a CYTEK AURORA flow cytometer (CYTEK Biosciences, Fremont, CA, USA). Data were analyzed using Flowjo software (Flowjo LLC, Ashland, OR, USA). Mouse antibodies against the following proteins were used for flow cytometry: CD4 (RM4-5), CD8a (53- 6.7), CD25 (PC61), CD44 (IM7), CD62L (MEL-14), Foxp3 (MF-14), ICOS (C398.4A), CXCR5 (L138D7), FAS (SA367H8), GL7 (GL7), CD138 (281-2), T-bet (4B10), GATA3 (16E10A23), TNFα (MP6-XT22), IFNγ (XMG1.2), IL-17A (TC11-18H10.1), CD45 (30 F11), CXCR4 (L276F12), PDPN (8.1.1), CD31 (390), IDO (mIOD-48), TGF-β1 (TW7-16B4), IL-10 (JES5-16E3), PD-L1 (10F.9G2), LPAM1 (DATK32), CCR7 (4B12), LFA1 (H155-78), CD49d (RI-2), VCAM1 (429 (MVCAM.A)), ICAM1 (YN1/1.7.4), CD62P (RMP-1), CXCR4 (L276F12), CXCR7 (8F11-M16), Granzyme B (QA18A28), Ki-67 (16A8), F4/80 (BM8), CD11c (N418), CD86 (GL-1), I-A/I-E (M5/114.15.2), XCR1 (ZET), CD172a (P84), and CD371 (927) were purchased from Biolegend. B220 (RA3 6B2), IgD (11 26c.2a), CD62E (10E9.6), and PSGL1 (2PH1), and CD80 (16-10A1) were purchased from BD Biosciences. Rorγt (AFKJS-9) was purchased from Thermo Fisher Scientific. CXCL12 (79018) was purchased from Invitrogen (Waltham, MA, USA). CCR2 (FAB5538F) was purchased from R&D. Human antibodies against the following proteins were used for flow cytometry: CD45 (2D1), PDPN (NC-08), CD31 (WM59), LAG3 (11C3C65), PD-L1 (29E.2A3), TGF-β1 (S20006A), IDO (eyedio), and IL-10 (JES3-9D7) were purchased from Biolegend. Arg1 (A1exF5) was purchased from Thermo Fisher Scientific.

In vivo migration assay of both CD11c$^+$ DCs and DsRed$^+$CD3$^+$ T cells with WT C57BL/6J mice and *Ccl19^{Cre}Ltbr$^{fl/fl}$* mice

CD3$^+$ T cells were isolated from the spleens of Ds-Red mice using EasySep™ Mouse T Cell Isolation Kit (STEMCELL Technologics, Vancouver, Canada, 19851). CD11c$^+$ DCs were isolated from the spleens and LNs of CD11c-GFP mice using CD11c MicroBeads UltraPure, mouse (Miltenyi Biotec, Bergisch Gladbach, Germany, 130-125-835), according to the manufacturer's protocol. For FRC-treated groups, WT-FRCs (2.0 x 10^5 cells per injection) were administrated intravenously four times, every other day. Twenty-four hours after the final FRC injection, 4.0 x 10^6 Ds-Red$^+$CD3$^+$ T cells and 2.5 x 10^6 GFP$^+$CD11c$^+$ DCs were injected subcutaneously into the left upper back of WT C57BL/6J mice and *Ccl19^{Cre}Ltbr$^{fl/fl}$* mice. Eight hours after the injections, the left axial and brachial LNs were collected and evaluated by flow cytometry.

Adoptive transfer of FRCs to naïve *Ccl19^{Cre}Ltbr$^{fl/fl}$* mice and visualization of conduit network

Naïve *Ccl19^{Cre}Ltbr$^{fl/fl}$* mice were injected i.v. with 1.0 x 10^5 isolated FRCs once a week for total 5 weeks. LNs were harvested and the structure of LNs was evaluated with IF staining. For the visualization of conduit network, a tracer dextran-FITC (40 kDa) (Thermo Fisher Scientific, D1845) was injected s.c. to WT C57BL/6J mice, *Ccl19^{Cre}Ltbr$^{fl/fl}$* mice, and FRCs-treated *Ccl19^{Cre}Ltbr$^{fl/fl}$* mice. Five minutes after injecting 2.5 μg FITC-dextran, the mice were euthanized and the LNs were harvested for immunofluorescence microscopy (2).

scRNA-seq

Cell preparation and sequencing methods were performed as described previously (3, 4). In order to assess the LNSCs at the transcriptional level after heart transplantation (day 8 after heart transplantation, anti-CD40L (250 μg, day 0)), we perform scRNA-seq using the 10X Genomics Chromium Single Cell Controller by the Institute of Genome Sciences of the University of Maryland in Baltimore. After enzymatic digestion of LNs harvested from C57BL/6 mice (8-12 weeks), CD45$^-$ cells were purified with the CD45

negative selection beads and were stained with anti-mouse CD45 before sorting for viable $CD45^-$ cells. For scRNA-seq, about 1.0×10^4 $CD45^-$ LNSCs were run on the 10X Chromium Controller (10X Genomics, Pleasanton, CA) to partition single cells into nanoliter-scale droplets containing uniquely barcoded beads and processed for sequencing library preparation using the Chromium Single Cell 3' Reagent Kit (v3 chemistry) (10x Genomics). Generated cDNA libraries were sequenced on a NovaSeq 6000 sequencing system at the Institute for Genome Sciences in the University of Maryland, Baltimore. Reads were demultiplexed, aligned to the GRCm38 mm10 assembly reference genome, and filtered; and cell barcodes and UMIs were quantified using the Cell Ranger 6.1 with default parameters (https://support.10xgenomics.com/single-cell-gene-expression/software/overview/welcome). 6.7×10^3 cells per sample were captured on the 10X Chromium chip. Raw data has been deposited in the NCBI's Gene Expression Omnibus (GEO) database GSE262918. Raw scRNA-seq matrix was analyzed using standard pipelines in Seurat V4.3.0. Low-quality cells were removed using the following options: 200 < nFeatures<7500, percent.mt < 20%. FindIntegrationAnchors and IntegrateData commands of Seurat (V 4.0.5) is used to integrate sample and correct the batch effect. Thirty principal components were used for dimension reduction and UMAP is used to visualize in the dimensionally reduced space. Clusters will be annotated based on public marker database. DEG was identified using the MAST test in Seurat. Only the genes with log2-fold change > 0.58, FDR < 0.05 are considered as significantly differential expressed. GO enrichment analysis of DEGs is done to identify the enriched pathways between conditions. Enriched GO terms were identified with FDR < 0.05.

Measurements of secreted CXCL12 from FRC

FRCs were passaged and incubated for 24 hours. Then, the medium was aspirated, and FRCs were washed with PBS twice. Fresh culture medium was filled into each well and treated with anti-LTβR agonist mAb (clone 3C8, AdipoGen, SanDiego, CA, USA, AG-20B-0041PF-C100) for 48 hours. Conditional mediums of FRC were harvested and centrifuged for the removal of cell debris. Cleared supernatant was stocked at -80 degree. Secreted CXCL12 was evaluated using Mouse CXCL12/SDF-1 ELISA Kit

(Proteintech, Rosemont, IL, USA, KE10049) according to the manufacturer's protocol. The absorbance was evaluated with a microplate reader (VersaMAX, San Jose, CA, USA).

Chemotaxis assay with conditional medium from cultured FRCs

$CD3^+$ T cells were isolated from mouse spleens using EasySep™ Mouse T Cell Isolation Kit. $CD3^+$ T cells (1.0 x 10^5 cells/well) were plated in the upper chambers of a 24-well transwell system (3 μm pore size; Corning) containing DMEM with 2% FBS. For groups treated with the CXCR4 antagonist, isolated T cells were pre-incubated with 1 μg/L AMD3100 (Selleck Chemicals, Houston, TX, USA, S8030) for 1 hour prior to plating in the upper chamber. Serum-free medium or conditional medium from cultured WT-FRCs or CXCL12 KO-FRCs were filled in the lower chambers. After the incubation for 4 hours, cells in lower chambers were collected and the number of migrated T cells were evaluated by flow cytometry gating on $CD4^+CD44^-CD62L^+$ (naïve $CD4^+$ T cells), $CD4^+CD44^+CD62L^-$ (effector $CD4^+$ T cells), and $CD4^+CD25^+Foxp3^+$ ($CD4^+$ Tregs).

Evaluation of effects of FRCs on T cell activation and differentiation from naïve T cells

For T cell activation assay, $CD3^+$ T cells were isolated from mouse spleens using EasySep™ Mouse T Cell Isolation Kit. WT C57BL/6J and *Ccl19*^{Cre}*Cxcl12*$^{fl/fl}$ FRCs (5.0 x 10^3 cells/well) were plated into 96 well plates. After the incubation for 24 hours, $CD3^+$ T cells (5.0 x 10^4 cells/well) were co-cultured with each FRCs. Anti-CD3e mAb (1 μg/m, clone 145-2C11, Thermo Fisher Scientific, 16-0031-82), anti-CD28 mAb (2 μg/mL, clone 37.51, Thermo Fisher Scientific, 14-0281-82), and IL-2 (10ng/mL, Biolegend, 575404) were added. Cytokine production was evaluated using quantitative real-time PCR (qPCR) after 48 hours of incubation, and T cell activation was assessed by flow cytometry after 96 hours of incubation.

For T cell differentiation assay, $CD4^+$ T cells were isolated from mouse spleens using the $CD4^+$ T Cell Isolation Kit, mouse (Miltenyi Biotec). 5.0 x 10^3 FRCs from WT C57BL/6J and *Ccl19*^{Cre}*Cxcl12*$^{fl/fl}$ mice were plated into 96 well plate. After the incubation for 24 hours, 5.0 x 10^4 $CD4^+$ T cells were co-cultured with each FRCs. For Th1 environment, anti-CD3e mAb (1 μg/mL, clone 145-2C11, Thermo Fisher Scientific), anti-CD28 mAb (2 μg/mL, clone 37.51, Thermo Fisher Scientific), IL2 (20ng/mL, Biolegend),

IL12 (20ng/mL, Biolegend, 577002) and anti-IL4 mAb (1µg/mL, clone 11B11, Thermo Fisher Scientific, 16-7041-81) were added at initiation of co-culture.

For Th2 environment, anti-CD3e mAb (1 µg/mL), anti-CD28 mAb (2 µg/mL), IL2 (20 ng/mL), IL4 (100ng/mL, Biolegend, 574302), anti-IFNγ mAb (1.0µg/mL, clone XMG1.2, Biolegend, 505801), and anti-IL12 mAb (0.5µg/mL, clone C18.2, Biolegend, 511801) were added at initiation of co-culture. For Th17 environment, anti-CD3e mAb (1 µg/mL), anti-CD28 mAb (2 µg/mL), TGF-β1 (1.0 ng/mL, Biolegend, 763102), IL6 (50 ng/mL, Biolegend, 575704), IL23 (5.0 ng/mL, Biolegend, 589002), anti-IL4 mAb (10 µg/mL, clone 11B11, Thermo Fisher Scientific), and anti-IFNγ mAb (10 µg/mL, clone XMG1.2, Biolegend) were added at initiation of co-culture. For Treg environment, anti-CD3e mAb (1 µg/mL), anti-CD28 mAb (2 µg/mL), TGF-β1 (10 ng/mL), and IL2 (10 ng/mL) were added at initiation of co-culture. After the incubation for 96 hours, T cell differentiations were evaluated by flow cytometry.

Evaluation of the inhibition of CXCR4 and G-protein coupled receptor on Th1 differentiation

5.0 x 10^3 FRCs from WT C57BL/6J mice were plated into a 96 well plate. After 24 hours of incubation, isolated $CD4^+$ T cells were co-cultured with FRCs. Each 5.0 x 10^4 $CD4^+$ T cells were pre-treated with DMOS, 1µg/L CXCR4 antagonist (AMD3100), or 500ng/mL Pertussis toxin (MedChemExpress, Monmouth Junction, NJ, USA, 516560) for 1 hour before starting the co-culture. After incubation under Th1 conditions for 96 hours, Th1 differentiation was evaluated by flow cytometry.

Synthesis and characterization of NPs

Poly(d,l-lactic-*co*-glycolic) acid-b-methoxy poly (ethylene glycol)-copolymer (PLGA-mPEG, MW 30,000:5,000 Da, 50:50 LA:GA (w:w)) and poly(d,l-lactic-*co*-glycolic) acid-b-poly(ethylene glycol)-maleimide (PLGA-PEG-MAL, MW 30,000:5,000 Da, 50:50 LA:GA (w:w)) were purchased from PolySciTech®, Akina Inc (West Lafayette, IN, USA, AK102 and AI110). PLGA-based NPs were synthesized using a water/oil/water (W/O/W) double emulsion method. PLGA-PEG (22.5 mg) and PLGA-PEG-MAL (2.5 mg) were dissolved in ethyl acetate. Mouse recombinant CXCL12 protein (SinoBiological, Wayne, PA, USA, 50025-MNAE) was prepared in Dulbecco's phosphate-buffered saline (DPBS,

Mediatech, Inc., Manassas, VA, USA), and sonicated for 30 s. The protein solution was emulsified dropwise into a 2% aqueous solution of poly(vinyl alcohol) (PVP, Sigma-Aldrich) under vigorous vortexing and then sonicated for 1 minute. The resulting emulsion was stirred for 2 hours to evaporate ethyl acetate. NPs were collected by ultracentrifugation at 21,000 rpm for 30 minutes (Beckman, Brea, CA, USA, L-80), and redispersed in DPBS. For the conjugation of MECA79 on the surface of NP, tris(2-carboxyethyl) phosphine hydrochloride (TCEP, 0.5 M, Sigma-Aldrich, 646547) was used to cleave disulfide bonds in MECA79 (1 mg/mL, NOVUS Biologicals, NB100-77673). Fifteen μg of MECA79 was incubated with 15 μL of TCEP for 15 min at room temperature. The reduced MECA79 was mixed with the NP suspension and incubated for 1 day at 4 °C. The NPs were subsequently collected by ultracentrifugation, washed, and redispersed in DPBS prior to use.

Hydrodynamic size of synthesized NPs was analyzed by a particle size analyzer (NanoBrook Omni, Nashua, NH, USA). The CXCL12-NP filtrate was analyzed by bicinchoninic acid (BCA) (Sigma-Aldrich) to calculate the loading efficiency of CXCL12 (defined as amount of CXCL12 loaded in NP/amount of initial CXCL12): 24.8%. Briefly, a calibration curve was generated by measuring the absorbance at 562 nm, which results from the complexation between CXCL12 and copper ions. The amount of CXCL12 loaded into the NPs was determined using this calibration curve.

Alexa 594-CXCL12 was synthesized by reacting CXCL12 with Alexa 594 NHS ester (Thermo Fisher Scientific, A20004) for 2 hours at room temperature. Excess unreacted dye was removed using a desalting column (Thermo Fisher Scientific, Zeba™, 7 kD MWCO, 89890). Alexa 594-CXCL12-NP was prepared with the identical procedure for CXCL12-NP as mentioned above.

Alexa594-CXCL12-MECA79-NP was incubated in 37 ^{o}C, and at predetermined time points, the aliquots were taken and centrifuged. The supernatants were measured by UV-vis spectroscopy to quantify the released amount of Alexa 594-CXCL12.

Evaluation of CXCL12-MECA79-NPs distribution

Aleca594-CXCL12 and Alexa594-CXCL12-MECA79-NPs were injected i.v. into skin transplant recipient mice on day 7 after transplantation. Each injection contained 100 μg of CXCL12 in 100 μL of PBS.

Twenty-four hours after injection, the mice were euthanized by carbon dioxide inhalation followed by cervical dislocation, and their DLNs were harvested. Ex vivo fluorescence imaging of the DLNs was performed using the iBOX explorer2 (UVP Ltd., Upland, CA, USA). MFI was acquired using the region of interest (ROI) function in ImageJ (National Institutes of Health, Bethesda, MD, USA). In addition, the harvested DLNs were embedded in OCT compound, and the distribution of injected CXCL12-MECA79-NPs was evaluated by IF.

References

1. Li X, et al. Lymph node fibroblastic reticular cells deposit fibrosis-associated collagen following organ transplantation. *J Clin Invest.* 2020;130(8):4182-94.
2. Novkovic M, et al. Topological Structure and Robustness of the Lymph Node Conduit System. *Cell Rep.* 2020;30(3):893-904 e6.
3. Li L, et al. Lymph node fibroblastic reticular cells preserve a tolerogenic niche in allograft transplantation through laminin alpha4. *J Clin Invest.* 2022;132(13).
4. Li L, et al. FRC transplantation restores lymph node conduit defects in laminin alpha4-deficient mice. *JCI Insight.* 2023;8(8).

Supplemental Figure 1

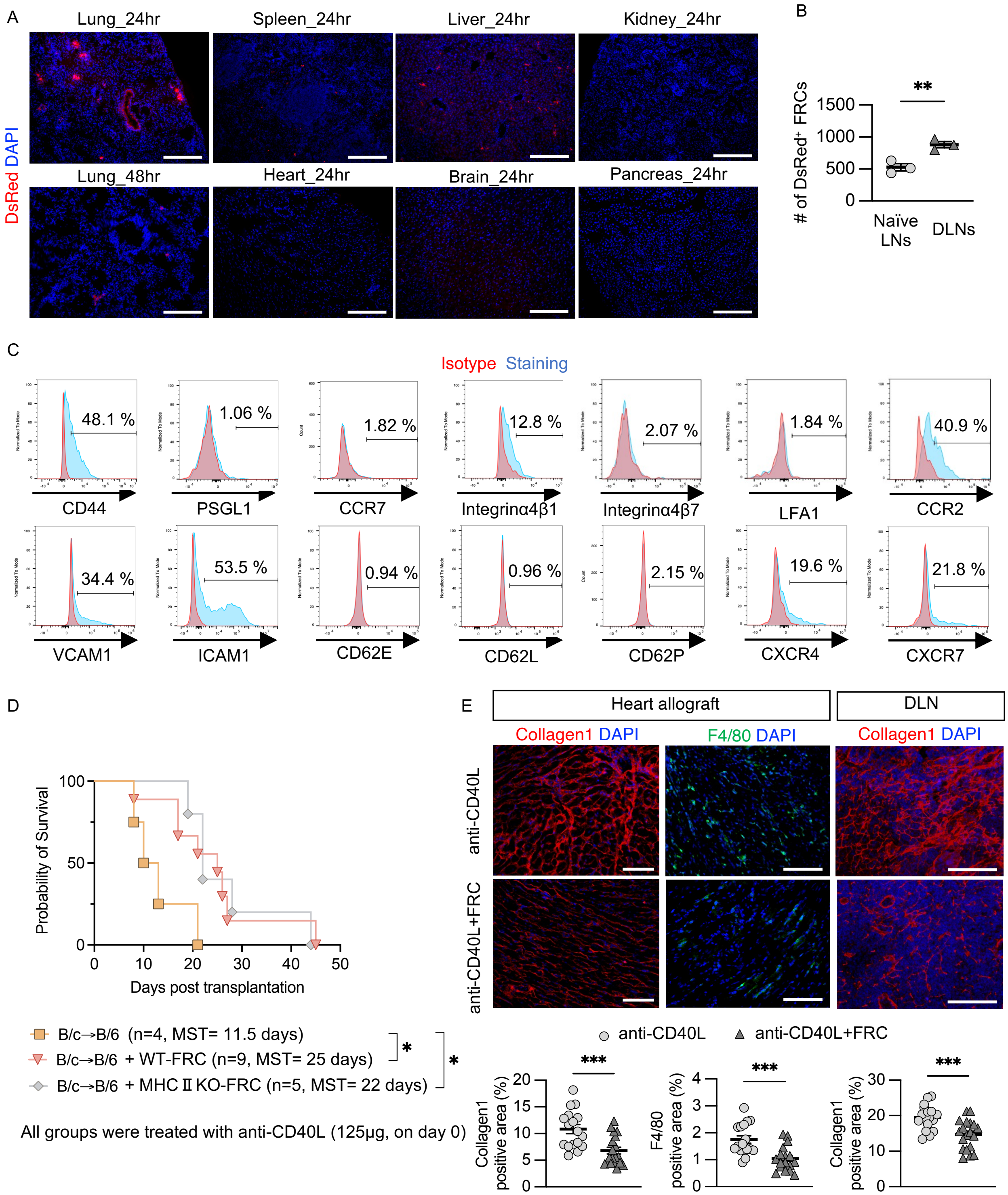

Supplemental Figure 1. Adoptive transfer of FRCs suppresses allograft rejection.
(**A**) Representative images of various organs from DsRed+ FRCs-injected mice 24 and 48 hours after the last FRC injection (n=3 mice/group). Scale bars: 200µm. (**B**) Comparison of the numbers of injected DsRed+ FRCs in naïve LNs and DLNs of heart allografts from WT C57BL/6J mice by flow cytometry (n=3 mice/group). (**C**) Expression of adhesion molecules and chemokine receptors on FRCs in naïve LNs by flow cytometry. (**D**) Comparison of heart allograft survival among various recipient groups (n=4-9 mice/group). (**E**) Representative images of heart allografts and DLNs (n=3-4 mice/group). Scale bars: 100µm. Log-rank test for graft survival. Student's t test for comparisons between two groups. Data presented as mean ± SEM. *$P < 0.05$, **$P < 0.01$, ***$P < 0.001$.

Supplemental Figure 2

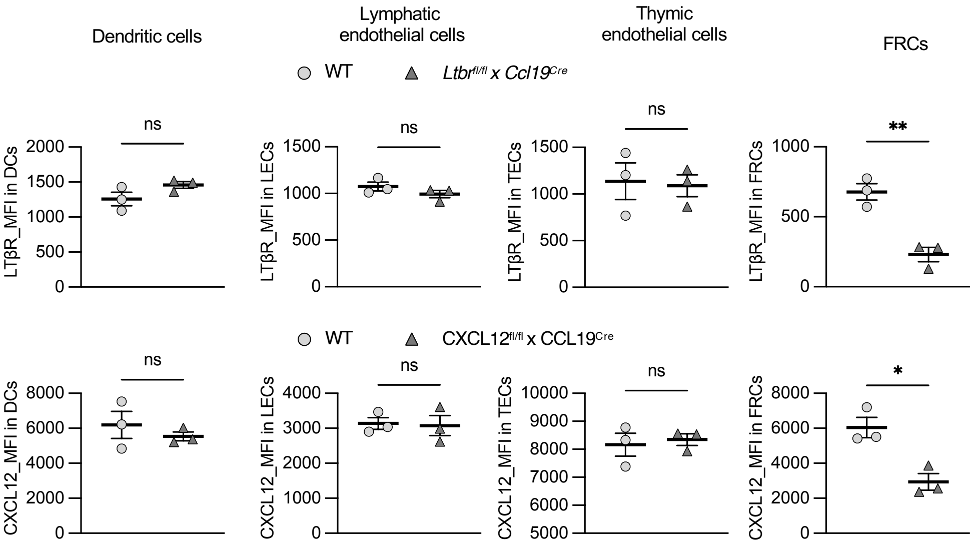

Supplemental Figure 2. Expression of LTβR and CXCL12 in FRCs and other cell types.
Comparison of LTβR and CXCL12 expression across different cell populations, including $CD11c^{+}$ dendritic cells (DCs) from the spleen, lymphatic endothelial cells ($CD45^{-}PDPN^{+}CD31^{+}$) and FRCs ($CD45^{-}PDPN^{+}CD31^{-}$) from LNs, and thymic endothelial cells ($CD45^{-}EpCAM^{+}$) from the thymus (n=3 mice/group). Student's t test for comparisons between two groups. Data presented as mean ± SEM. $^{*}P < 0.05$, ns: not significant.

Supplemental Figure 3

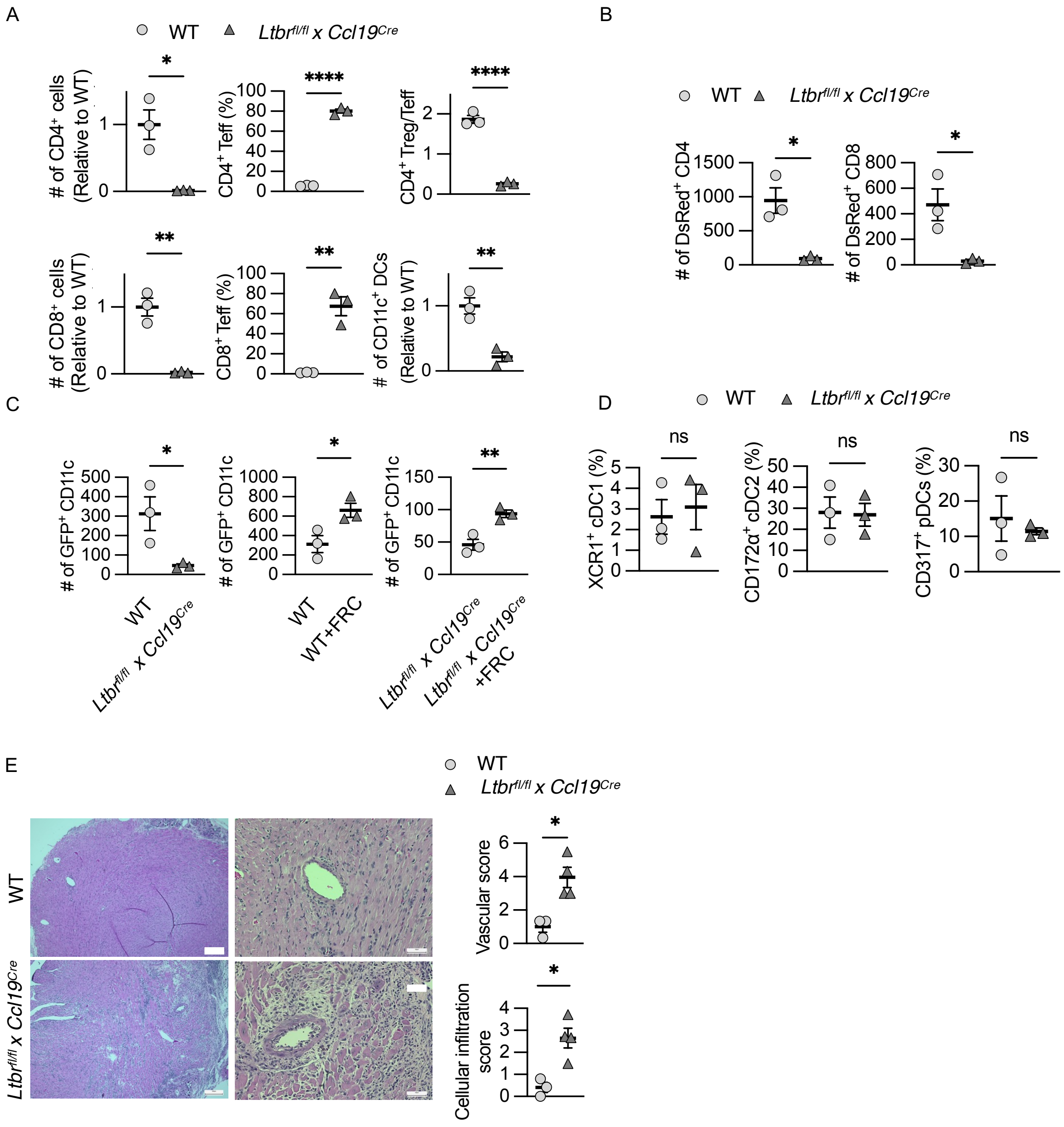

Supplemental Figure 3. LN analysis of WT C57BL/6J and *Ccl19^Cre^Ltbr^fl/fl^* mice

(**A**) Comparison of the numbers of CD4⁺ T cells, CD8⁺ T cells, and CD11c⁺ DCs in naïve LNs. Comparison of the percentages of Teff, CD4⁺ Treg/Teff in naïve LNs (n=3 mice/group). (**B-C**) In vivo migration assay of subcutaneously injected DsRed⁺ T cells (**B**) and GFP⁺CD11c⁺ DCs (**C**) in DLNs (n=3 mice/group). DC homing to DLNs was also assessed following FRC treatment and no FRC treatment. (**D**) Comparison of the percentage of cDC1s, cDC2s, and pDCs in LNs (n=3 mice/group). (**E**) Representative images of H&E-stained heart allografts. Scale bars: 50 µm. Student's t test for comparisons between two groups. Data presented as mean ± SEM. *P < 0.05, **P < 0.01, ****P < 0.0001, ns: not significant.

Supplemental Figure 4

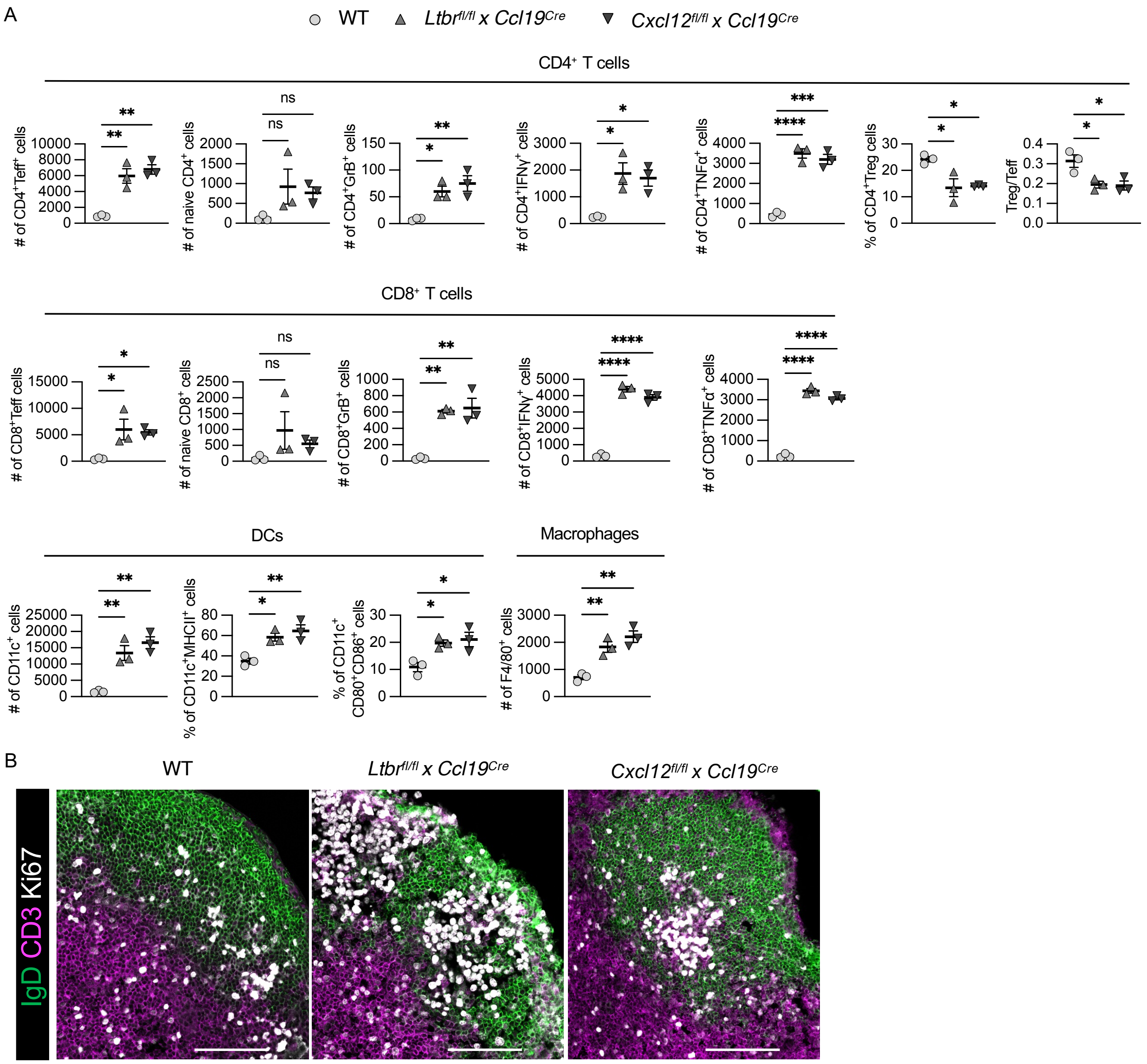

Supplemental Figure 4. Comparison of infiltrated immune cells in heart allografts and germinal centers in DLNs.
(**A**) Flow cytometry analysis of heart allografts (n=3 mice/group). (**B**) Representative images of CD3, IgD, and Ki67-stained DLNs (n=3 mice/group). Scale bars: 100 µm. One-way ANOVA with Tukey's multiple comparisons test for multiple comparisons. Data presented as mean ± SEM. *P < 0.05, **P < 0.01, ***P < 0.001, ****P < 0.0001, ns: not significant.

Supplemental Figure 5

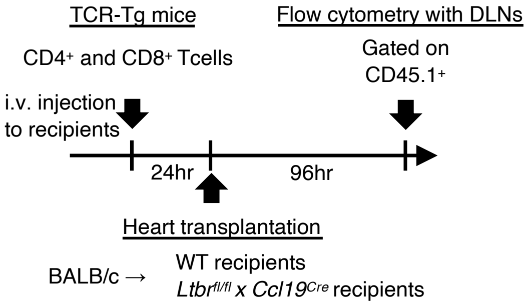

Supplemental Figure 5. Schematic of heart transplantation with adoptive transfer of alloantigen-specific TCR Tg T cells.

Supplemental Figure 6

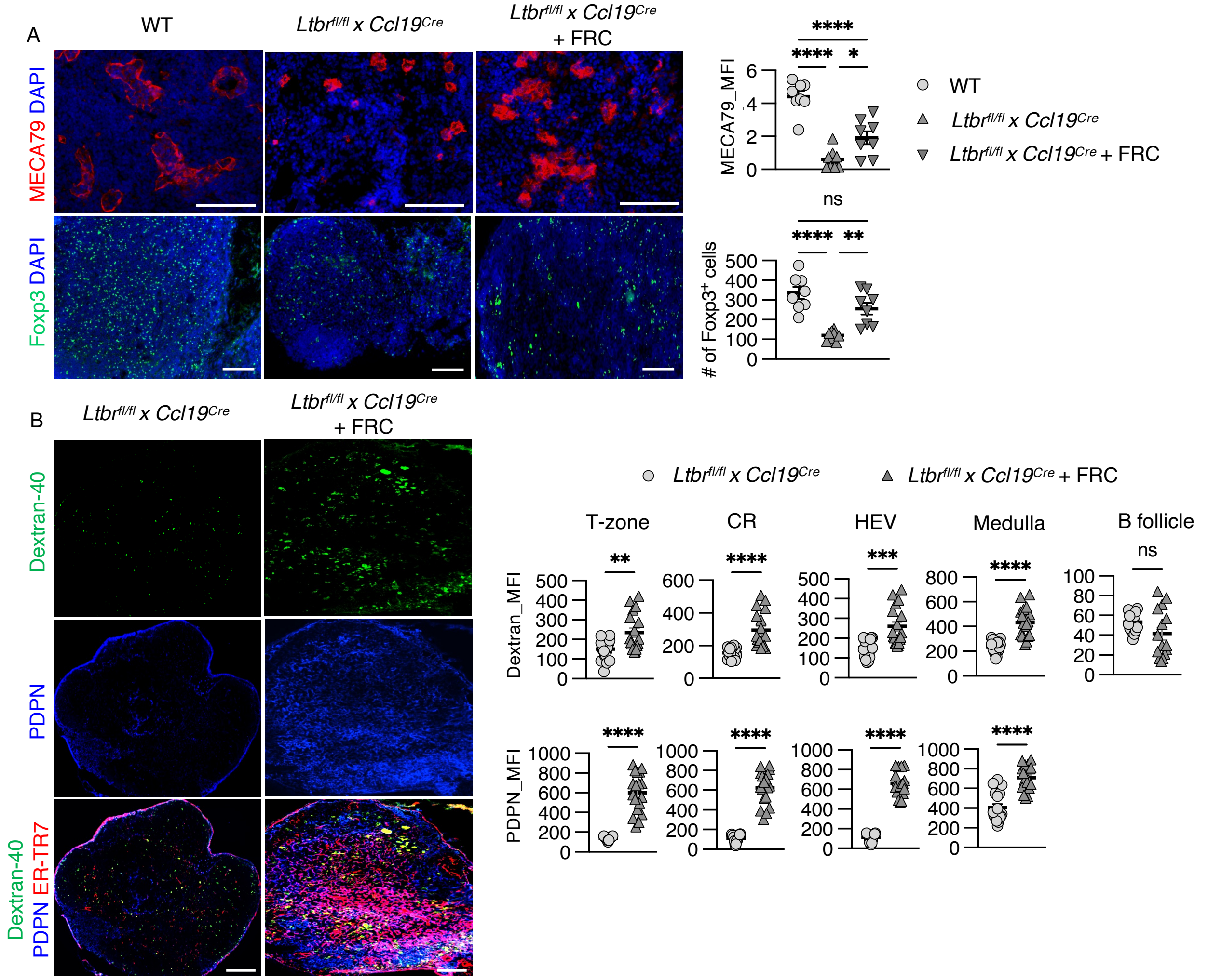

Supplemental Figure 6. Injection of WT-FRCs restores conduit network in *Ccl19^Cre^Ltbr^fl/fl^* mice

(**A**) Representative images and quantification of MECA79 and Foxp3-stained LNs in WT C57BL/6J, *Ccl19^Cre^Ltbr^fl/fl^*, FRCs-injected *Ccl19^Cre^Ltbr^fl/fl^* mice (n=3 mice/group). Scale bars: 100 μm. (**B**) Representative images and quantification of dextran-40 and PDPN in LNs from *Ccl19^Cre^Ltbr^fl/fl^* and FRCs-injected *Ccl19^Cre^Ltbr^fl/fl^* mice (n=3 mice/group). Scale bars: 100 μm. Student's t test for comparisons between two groups. One-way ANOVA with Tukey's multiple comparisons test for multiple comparisons. Data presented as mean ± SEM. *P < 0.05, **P < 0.01, ***P < 0.001, ****P < 0.0001, ns: not significant.

Supplemental Figure 7

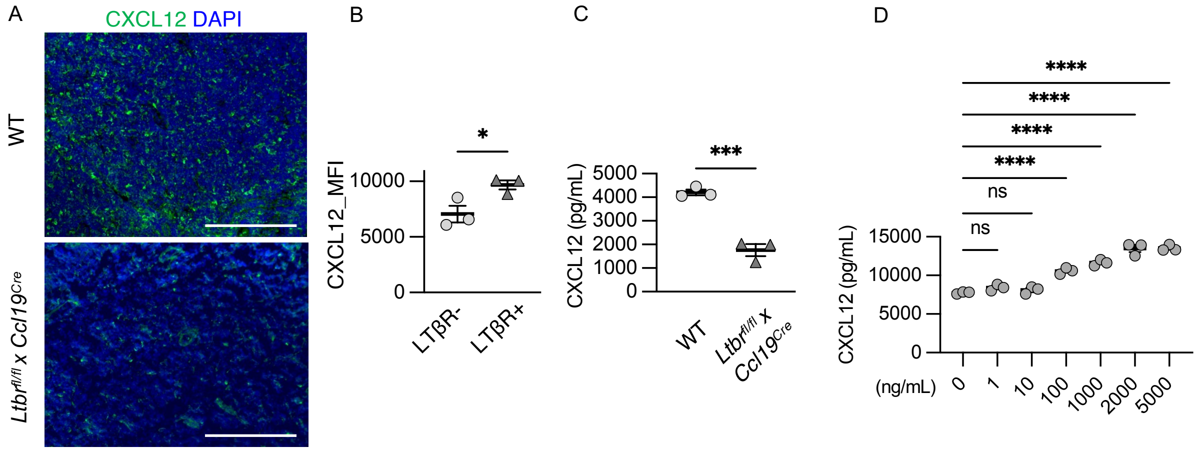

Supplemental Figure 7. Relationship between LTβR and CXCL12 in FRCs

(**A**) Representative images of CXCL12 in LNs from naïve WT C57BL/6J mice and *Ccl19*^{Cre}*Ltbr*$^{fl/fl}$ mice. Scale bar: 200 μm. (**B**) Comparison of CXCL12 expression in FRCs isolated from WT C57BL/6J mice by flow cytometry (n=3/group). (**C**) Comparison of CXCL12 expression in supernatants of FRCs 24 hours after incubation (n=3/group). (**D**) Comparison of CXCL12 expression in supernatants of FRCs stimulated with anti-LTβR agonist mAbs for 48 hours (n=3/group). Student's t test for comparisons between two groups. One-way ANOVA with Tukey's multiple comparisons test for multiple comparisons. Data presented as mean ± SEM. *P < 0.05, ***P < 0.001, ****P < 0.0001, ns: not significant.

Supplemental Figure 8

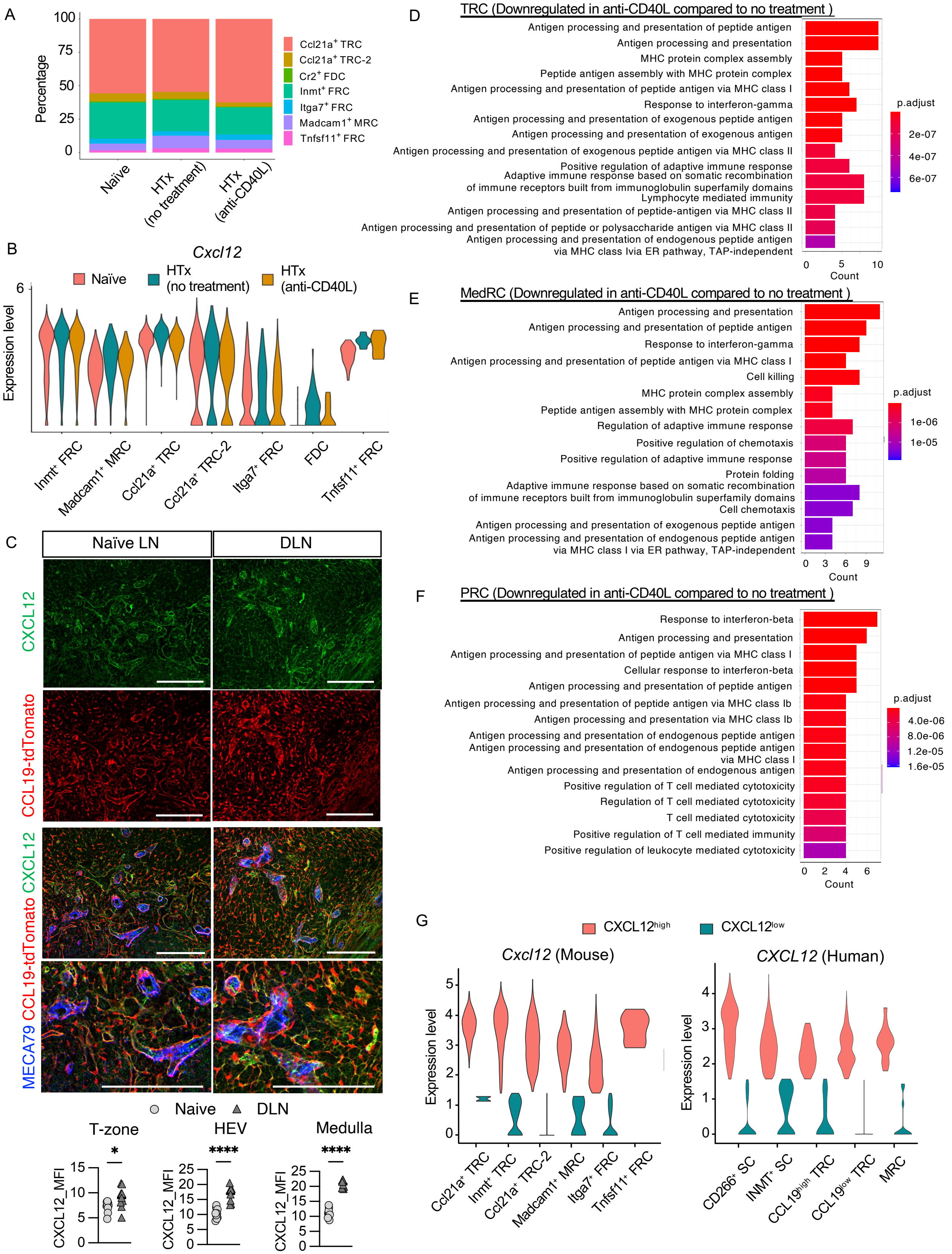

Supplemental Figure 8. scRNA-seq analysis of LNSCs from mouse and human LNs. (**A**) Percentage of the FRC clusters in naïve LNs and DLNs. (**B**) Violin plots of *Cxcl12* expression in FRCs. (**C**) Representative images and quantification of CXCL12 and MECA79-stained LNs in *Ccl19*^{Cre}tdTomato mice (n=3 mice/group). Scale bars: 200 µm. (**D-F**) Top 15 over-represented ontology pathway in TRCs (**D**), MedRCs (**E**), and PRCs (**F**) based on downregulated genes in anti-CD40L group compared to no treatment group. (**G**) Expression of *CXCL12* amongst FRC subsets. One-way ANOVA with Tukey's multiple comparisons test for multiple comparisons. Data presented as mean ± SEM. *P < 0.05, **P < 0.01, ns: not significant. HTx: Heart transplantation.

Supplemental Figure 9

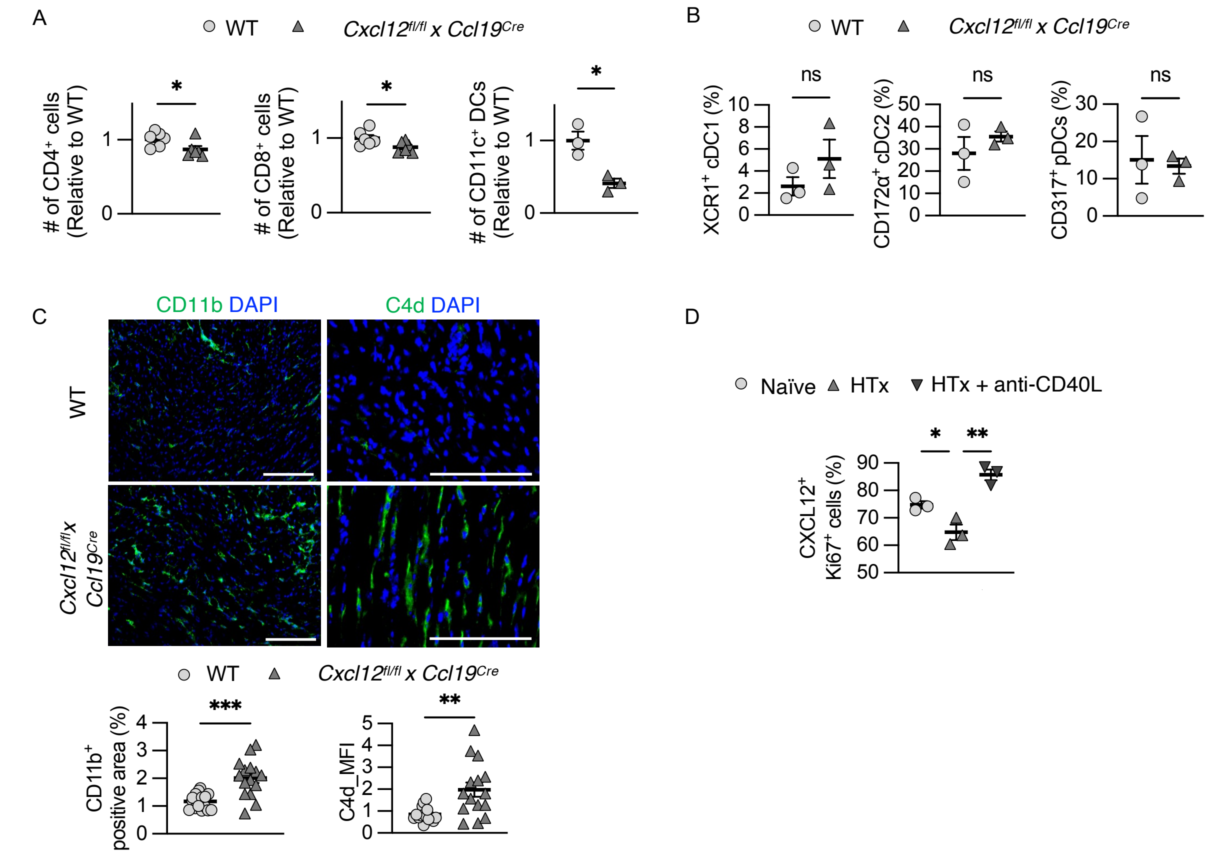

Supplemental Figure 9. LN analysis of WT C57BL/6J and *Ccl19^Cre^Cxcl12^fl/fl^* mice

(**A**) Comparison of the numbers of T cells and DCs in naïve LNs (n=3-6 mice/group). (**B**) Comparison of the percentage of cDC1s, cDC2s, and pDCs in LNs (n=3 mice/group). (**C**) Representative images and comparison of CD11b and C4d-stained heart allografts (n=5 mice/group). Scale bar: 200 μm. (**D**) Comparison of the percentages of CXCL12+Ki67+ FRCs (n=3 mice/group). Student's t test for comparisons between two groups. Data presented as mean ± SEM. *P < 0.05, **P < 0.01, ***P < 0.001. HTx: Heart transplantation.

Supplemental Figure 10

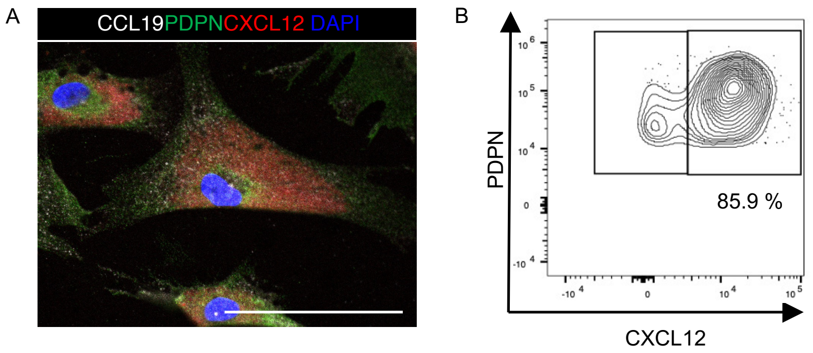

Supplemental Figure 10. Expression of CXCL12 in cultured FRCs from human LNs
(**A**) Representative image of CXCL12-stained FRCs with co-staining of CCL19 and PDPN. Scale bars: 100μm. (**B**) Gating of CXCL12^{low} and CXCL12^{high} FRCs in CD45^{-}PDPN^{+}CD31^{-} population.

Supplemental Table 1

Mouse *IFNγ*-F: 5′- TTGAGGTCAACAACCCACAG -3′

Mouse *IFNγ*-R: 5′- ATCAGCAGCGACTCCTTTTC -3′

Mouse *GranzymeB*-F: 5′-CCTCCTGCTACTGCTGAC -3′

Mouse *GranzymeB*-R: 5′-GTCAGCACAAAGTCCTCTC -3′

Mouse *Gapdh*-F: 5′- AGGTCGGTGTGAACGGATTTG -3′

Mouse *Gapdh*-R: 5′- TGTAGATGTAGTTGAGGTCA-3′
